# Evidence That Dmrta2 Acts through Repression of *Pax6* in Cortical Patterning and Identification of a Mutation Impairing DNA Recognition Associated with Microcephaly in Human

**DOI:** 10.1523/ENEURO.0377-24.2025

**Published:** 2025-06-13

**Authors:** Xueyi Shen, Jithu Anirudhan, Ambrin Fatima, Estelle Plant, Tünde Szemes, Zélie Bouveret, Marc Keruzore, Sadia Kricha, Xinsheng Nan, Alba Sabaté San José, Samuel Bianchin, Bérénice Veraghen, Louis-Paul Delhaye, Bilal Ahmad Mian, Lubaba Bintee Khalid, Farhan Ali, Hijab Zahra, Asmat Ali, Mathias Toft, Marc Dieu, Younes Achouri, Meng Li, Patricia Renard, Carine Van Lint, Coralie Poulard, Zafar Iqbal, Eric J. Bellefroid

**Affiliations:** ^1^Laboratory of Developmental Genetics, Department of Molecular Biology and ULB Neuroscience Institute (UNI), Université Libre de Bruxelles, Gosselies 6041, Belgium; ^2^Department of Biological and Biomedical Sciences, Aga Khan University, Karachi 74800, Pakistan; ^3^Service of Molecular Virology, Department of Molecular Biology and ULB-Cancer Research Centre, Université Libre de Bruxelles, Gosselies 6041, Belgium; ^4^INSERM U1052, CNRS UMR5286, Université Claude Bernard Lyon1, Centre de Recherche en Cancérologie de Lyon, Lyon F-69000, France; ^5^Neuroscience and Mental Health Research Institute, School of Medicine, Cardiff University, Cardiff CF24 4HQ, United Kingdom; ^6^Department of Paediatrics and Child Health, Aga Khan University, Karachi 74800, Pakistan; ^7^Department of Neurology, Oslo University Hospital, Oslo 0424, Norway; ^8^Institute of Clinical Medicine, University of Oslo, Oslo 0424, Norway; ^9^MaSUN-Proteomic Facility, Université de Namur, Namur 5000, Belgium; ^10^Transgene Technology Platform TRSG, Université catholique de Louvain, Leuven 1348, Belgium

**Keywords:** cerebral cortex, microcephaly, neural progenitor, neurogenesis, patterning, transcription factor

## Abstract

Dmrta2 (also designated Dmrt5) is a transcriptional regulator expressed in cortical progenitors in a caudomedial^high^/rostrolateral^low^ gradient with important roles at different steps of cortical development. Dmrta2 has been suggested to act in cortex development mainly by differential suppression of *Pax6* and other homeobox transcription factors such as the ventral telencephalic regulator *Gsx2*, which remains to be fully demonstrated. Here we have addressed the epistatic relation between Pax6 and Dmrta2 by comparing phenotypes in mutant embryos or embryos overexpressing both genes in various allelic combinations. We show that Dmrta2 cooperates with Pax6 in the maintenance of cortical identity in dorsal telencephalic progenitors and that it acts as a transcriptional repressor of *Pax6* to control cortical patterning. Mechanistically, we show that in P19 cells, Dmrta2 acts as a DNA binding-dependent repressor on the *Pax6 E60* enhancer and that a point mutation that affects its DNA binding properties identified in a consanguineous family leads to agenesis of the corpus callosum, pachygyria, and the absence of the cingulate gyrus. Finally, we provide evidence that Dmrta2 binds components of the NuRD repressor complex and interacts with zinc finger proteins such as Zfp423. Together, our results highlight the importance and conserved function of Dmrta2 in cortical development and provide novel insights into its mechanism of action.

## Graphical abstract

**Figure:**
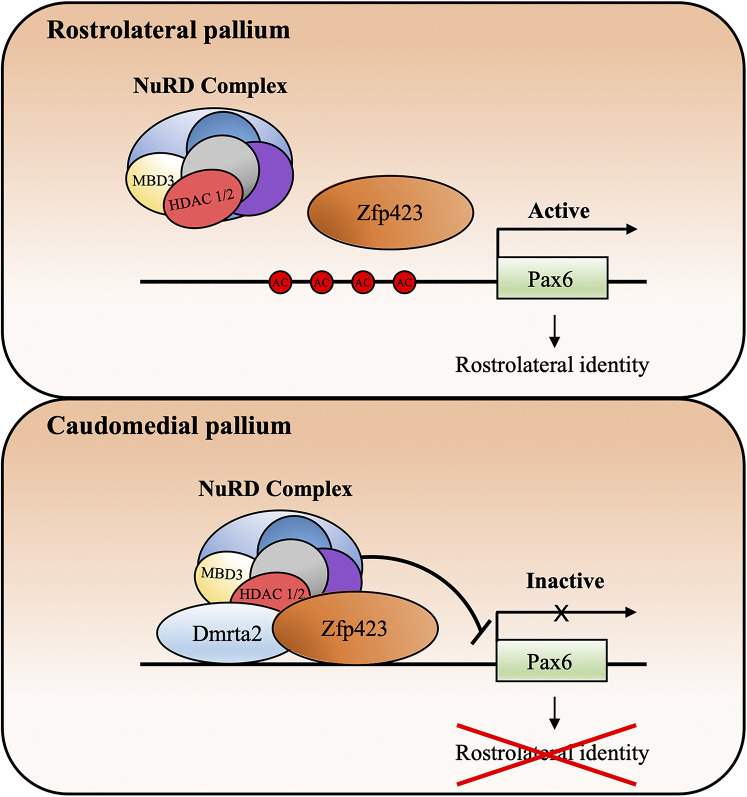
Visual Abstract

## Significance Statement

Corticogenesis is controlled by an array of transcription factors that coordinate neural progenitor self-renewal and differentiation to generate correct cortical cell number and diversity. However, how this complex array of transcription factors works in concert to regulate this process remains largely unknown. Here we provide insights into the mechanism of action of the transcription factor Dmrta2 by demonstrating that it cooperates with Pax6 to define the pallium-subpallium boundary and acts by repressing it to control cortical patterning. Our data also reveal that Dmrta2 interacts with components of the NuRD repressor complex and with the Zfp423 zinc finger protein and that a point mutation that affects its DNA binding causes cortical abnormalities in human, further highlighting its importance in cortex development.

## Introduction

Balancing neural progenitor self-renewal and differentiation is essential for generating cells in correct numbers and diverse types during neural development. During cerebral cortex development, neurogenesis is tightly regulated by a complex array of transcription factors (TFs). Many of these TFs are expressed in graded patterns along the rostral/caudal (R/C) and dorsal/ventral (D/V) axes of the developing ventricular zone (VZ) of the pallium (dorsal telencephalon) under the control of signals produced by localized signaling centers located at the periphery of the cortical primordium (Cadwell et al., 2019; Tole and Hébert, 2020; Ypsilanti et al., 2021). How this host of TFs expressed in gradients in progenitors orchestrates together their proliferation and differentiation to build the cerebral cortex remains today an important unanswered question.

Among the genes coding for cortical TFs is the zinc finger *Dmrta2* gene, also named *Dmrt5*, which is expressed in a caudomedial^high^/rostrolateral^low^ gradient in the developing cortical VZ. In mice lacking *Dmrta2*, the telencephalic vesicle size is decreased due to premature differentiation of progenitors (Young et al., 2017). The hippocampus and the cortical hem, one of the major telencephalic patterning centers, and the caudal neocortical area are strongly reduced (Konno et al., 2012; Saulnier et al., 2013). The additional knock-out of the related *Dmrt3* gene in mice lacking *Dmrta2* leads to a more severe phenotype with cortical progenitors expressing ventral telencephalic markers such as *Gsx2* (Desmaris et al., 2018). Conditional deletion of *Dmrta2* leaving cortical hem intact also results in a similar phenotype, suggesting that *Dmrta2* controls the patterning of the cortex independently of its influence on the hem. Conversely, in *Dmrta2* overexpressing mice, the caudal area expands while rostral ones are reduced (De Clercq et al., 2018).

Mechanistically, Dmrta2 has been shown to bind to telencephalic enhancers of the *Pax6* and *Gsx2* loci and repress their expression (Konno et al., 2019). While both *Dmrta2* and *Pax6* contribute to telencephalic dorsoventral patterning by repressing ventral telencephalic-specific genes, *Pax6* within cortical progenitors regulates in an opposite manner to *Dmrta2* patterning and arealization of the neocortex (Ypsilanti and Rubenstein, 2016). Although *Pax6* effects are context and dose dependent, it can promote neuronal differentiation while *Dmrta2* promotes the maintenance of neural progenitors during neurogenesis (Manuel et al., 2015; Young et al., 2017). Therefore, it has been suggested that *Dmrta2* acts through the repression of *Gsx2* to define the dorsal telencephalic compartment and through the repression of *Pax6* to determine positional information within cortical progenitors (Konno et al., 2019). First evidence for the hypothesis that *Dmrta2* acts through *Pax6* in cortex development has been obtained in *Dmrta2* loss of function experiments using electroporated siRNA, in the context of its opposite function to *Pax6* in the control of the differentiation of progenitors. Results obtained showed that the double knockdown of *Dmrt3* and *Dmrta2* upregulates the expression of proneural genes such as *Neurog2* or *Neurod1* that act as the primary initiators of neuronal differentiation and are directly activated by *Pax6*, while the additional knockdown of *Pax6* in *Dmrt3*; *Dmrta2* double knockdown rescued the neurogenic phenotype (Konno et al., 2019).

In this study, we compared phenotypes of mutant embryos for both *Dmrta2* and *Pax6* in various allelic conditions and double conditional transgenics overexpressing both *Dmrta2* and *Pax6* with transgenics overexpressing only *Dmrta2* to understand how *Dmrta2* and *Pax6* function together in cortex development. We then performed reporter assays in P19 teratocarcinoma cells and rapid immunoprecipitation followed by mass spectrometry of endogenous protein (RIME) experiments to approach the mechanism of action of Dmrta2. We also examined in human a novel case of microcephaly with a point mutation in *DMRTA2*. Together, our results demonstrate the importance of Dmrta2 and Pax6 interactions in the positional information of cortical progenitors and provide first insights into its mechanism of action.

## Materials and Methods

### Ethics statement

The animal experimental procedures were approved by the CEBEA (Comité d’éthique et du bien-être animal) of the IBMM (Institut de Biologie et de Médecine Moléculaires) -ULB and conformed to the European guidelines on the ethical care and use of animals.

### Animal lines and genotyping

The mid-day of the vaginal plug discovery was termed embryonic day 0.5 (E0.5), and the first 24 h (h) after birth was postnatal day (P) 0. Mice were provided *ad libitum* with standard mouse lab pellet food and water and housed at room temperature (RT) with a 12 h light/dark cycle.

*Dmrta2^+/−^* and *Dmrta2^Tg/+^* (De Clercq et al., 2018), *Pax6^sey/+^* (Hill et al., 1991), and *BAT-gal* (Maretto et al., 2003) mice were maintained in a C57Bl/6 background. *Pax6^Tg/+^* (Berger et al., 2007) were in an FVB/N background. The *Dmrta2* knock-in (*Dmrta2^2xHA^*) mouse line was generated using CRISPR/cas9 editing. A crRNA was designed using CRISPRdirect (http://crispr.dbcls.jp/) with the intent to produce a double-strand break in the second exon of *Dmrta2*, 5′ from the ATG codon. 2.4 pmol/µl of annealed crRNA (10 nmol, Integrated DNA Technologies, 5′-TCCAGCCATGGAGCTGCGCT-3′) and the tracrRNA (Integrated DNA Technologies, catalog #1072534) were injected into fertilized egg with 100 ng/μl of recombinant Cas9 protein (Integrated DNA Technologies) and 10 ng/μl of a single single-stranded DNA oligonucleotide template with homology arms to the targeted region and containing a 2XHA sequence (Integrated DNA Technologies, Megamer ssDNA Fragment: 5′-CTTAAGCCTAGTGACTGTATTACCCTGACTCAGGTATCCCCACTTTCCCCACGCTAGGTCCAGCCATGGAGTATCCCTATGACGTCCCGGACTATGCAGGATCCTATCCATATGACGTTCCAGATTACGCTGAGCTGCGCTCGGAGCTGCCCAGCGTGCCCGGTGCGGCGACAGCAGCAGCGACAGCGACGGGACCACCC-3′, with crRNA sequence underlined and *Dmrta2* ATG initiation codon in bold) into the pronucleus of B6D2F2 zygotes. Viable two-cell stage embryos were transferred to pseudopregnant CD1 females. Founder mice with the modified *Dmrta2^2xHA^* allele were screened for appropriate integration of the transgenic sequences at the knock-in region as well as for the presence of predicted (http://crispr.dbcls.jp/) potential off-targets followed by PCR followed by Sanger sequencing of the PCR products (Genewiz). They were then crossed with C57BL/6J. Germline transmission of the selected Dmrta2^2xHA^ founder mouse was then validated by PCR. Selected founders were then maintained in this background during at least three generations before to be used in this study.

The *Dmrta2* null and WT alleles were detected by PCR using primers Fwd: 5′-CGAATCTTTCGGACACTGTAGA-3′; Rev WT: 5′-CCAGAC CCTCAAGCACTCAA-3′; Rev KO: 5′-AGCGCCTCCCCTACCCGGTA-3′. The *Dmrta2^Tg^* allele was detected by PCR using primers Fwd Rosa26 5′-AAACTGGCCCTTGCCATTGG-3′; Fwd eGFP 5′-AACGAGAAGCGCGATCACAT-3′; Rev Rosa26 5′-GTCTTTAATCTACC TCGATGG-3′. The *Pax6^sey^* mutated allele was detected by high-resolution DNA melting analysis (HRMA; Reed et al., 2007) using primers 5′-AGGGGGAGAGAACACCAACT-3′ and 5′-CATCTGAGCTTCATCCGAGTC-3′. The *Dmrta2^2XHA^* allele was detected by PCR using primers inside the inserted 2XHA sequence (5′-TATCCCTATGACGTCCCGGAC-3′ and 5′-AGCGTAATCTGGAACGTCATAT-3′) and flanking the inserted 2XHA sequence (5′-TTCCAGTTCGTTTCCCCAGCA-3′ and 5′-GTACTTCTCCGCTGCCCTCAA-3′). As previous studies did not reveal any differences in *Dmrta2* nor *Pax6* gene expression between sexes, nonsex-determined embryos have been used in all our analyses.

### Immunofluorescence and in situ hybridization

For immunofluorescence (IF), dissected brains of embryos (E12.5) were fixed for 2 h at 4°C in 4% paraformaldehyde (PFA)/phosphate-buffered saline (PBS). Tissues were then rinsed with ice-cold PBS and cryopreserved by overnight (O/N) incubation in 30% sucrose/PBS O/N, subsequently embedded in gelatin (7.5% gelatin, 15% sucrose/PBS), and sectioned in 14 μm cryostat sections. Sections were then rinsed in PBS, permeabilized, and blocked in a solution of PBS with 0.3% Triton X-100 containing 10% goat serum for at least 2 h at RT and then incubated with primary antibodies diluted in blocking serum O/N, at 4°C. Slides were incubated in secondary antibodies diluted in PBST for 2 h at RT. The following primary antibodies were used: rabbit anti-Dmrta2 (1:1,000; De Clercq et al., 2018), rabbit anti-Flag (1:500, Sigma, catalog #F3165), rabbit anti-HA (1:500, Abcam, catalog #ab9110), and mouse anti-Zfp423 (1:500, Santa Cruz Biotechnology, catalog #sc-393904). The following secondary antibodies were used: anti-mouse Alexa Fluor 488 (1:400, Invitrogen, catalog #A-11017), anti-rabbit Alexa Fluor 488 (1:400, Invitrogen, catalog #A-11008), anti-mouse Alexa Fluor 594 (1:400, Invitrogen, catalog #A-11005), and anti-rabbit Alexa Fluor 594 (1:400, Invitrogen, catalog #A-11012). Sections were counterstained with DAPI. Images were acquired with a Carl Zeiss LSM 710 confocal microscope using Zen-Black software or Nikon A1R gallium arsenide phosphide inverted confocal microscope and processed using ImageJ and Photoshop software.

In situ hybridization (ISH) on whole brains or sections were processed with digoxigenin (Dig)-labeled riboprobes as described (Schaeren-Wiemers and Gerfin-Moser, 1993; Wilkinson and Nieto, 1993). The brains of embryos (E11.5, E12.5, and E15.5) and newborn mice (P7) were fixed in 4% PFA/PBS at 4°C, O/N. For ISH on sections, the brains were infused in 30% sucrose/PBS, O/N, and frozen in gelatin (7.5% gelatin, 15% sucrose/PBS). Then, 20 μm cryostat sections were collected. For ISH on whole mount, the brains were dehydrated by rinsing 15 min (min) in successive baths of MetOH-PTw (25, 50, 75, and 100%) and stored at −20°C. The probes were generated from the following previously described cDNA clones: *Dmrta2* (Saulnier et al., 2013), *Gsx1* and *Gsx2* (Toresson et al., 2000), *Pax6* (Hamasaki et al., 2004), *Rorβ* (De Clercq et al., 2018), *Wnt3a* (Monuki et al., 2001), *Wnt8b* (Lee et al., 2000), and *Zfp423* (Masserdotti et al., 2010). Images were acquired with an Olympus SZX16 stereomicroscope and an XC50 camera, using the Imaging software cellSens.

Quantification of the dorsal surface area of the cortical hemisphere of E18.5 embryos was obtained by taking measurements from images of whole brains. The surface areas of the primary motor, sensorimotor, and visual neocortex in P7 brains were measured by taking measurements from the images and outlining the corresponding regions with specific probes as indicated in the text. The results are presented as the ratio of the M1, S1, and V1 areas relative to the total dorsal surface area of the cortex. Micrographs were taken with an Olympus SZX16 stereomicroscope. Measurements were done using the Imaging software cellSens. All quantified data are expressed as mean values ± standard deviation (SD). Significance tests were performed using a two-tailed Student's *t* test; *p* values <0.05 were regarded as statistically significant. Each experiment was repeated on at least four biological samples for each genotype.

### Whole-mount X-gal staining of embryos

Embryos were fixed in 4% PFA for 1 h at RT and rinsed in detergent solution (2 mM MgCl_2_, 0.01% sodium deoxycholate, 0.02% NP-40 diluted in 0.1 M, pH 7.3, phosphate buffer). Embryos were then incubated at 37°C in a staining solution composed of detergent solution with 5 mM K_3_Fe(CN)_6_, 5 mM K_4_Fe(CN)_6_, and 1 mg/ml of X-gal. Before clearing embryos were progressively dehydrated in increasing concentrations of MetOH. After several washes in 100% MetOH, embryos were incubated in a solution of MetOH:BABB (1:1) before being incubated and stored in 100% BABB. BABB is made of one part benzyl alcohol for two parts of benzyl benzoate.

### RNA isolation and RT-qPCR

The cortex of embryos was dissected in RNAase-free cold 1× PBS and then immediately frozen at −80°C. RNA extraction was performed using the Monarch Total RNA Miniprep Kit (New England Biolabs, catalog #T2010s) according to the manufacturer's protocol. cDNA was synthesized starting from 1–2 μg of total RNA using the iScript cDNA Synthesis Kit (Bio-Rad, catalog #1708891). RT-qPCRs were carried out with the Luna Universal qPCR Master Mix (New England Biolabs, catalog #M3003) using the StepOne Plus Real-Time PCR system (Applied Biosystems). RT-qPCRs primers used were as follows: *Pax6* (5′-AGGGCAATCGGAGGGAGTAA-3′ and 5′-CAGGTTGCGAAGAACTCTGTTT-3′); *Emx2* (5′-GTCCACCTTCTACCCCTGG-3′ and 5′-CCACCACGTAATGGTTCTTCTC-3′); *Lhx2* (5′-TGGCAGCATCTACTGCAAAG-3′ and 5′-TGTGCATGTGAAGCAGTTGA-3′); *Wnt3a* (5′-CAGGAACTACGTGGAGATCATGC-3′ and 5′-CATGGACAAAGGCTGACTCC-3′); *Wnt8b* (5′-CAGCTCTGCTGGGGTTATGT-3′ and 5′-CTGCTTGGAAATTGCCTCTC-3′) and *GAPDH* (5′-GTGAAGGTCGGTGTGAACG-3′ and 5′-AGGGGTCGTTGATGGCAACA-3′) used as a reference gene for normalizing gene expression results. Error bars show the standard deviations of at least three independent experiments. To detect by RT-PCR the *Dmrta2* tagged allele in dissected cortices of *Dmrta2^2xHA^* transgenics, primers 5′-TTCCAGTTCGTTTCCCCAGCA-3′ and 5′-GTACTTCTCCGCTGCCCTCAA-3′ were used.

### Plasmids

For reporter assays, to generate the pGL4.23-*E60*- tk-luc reporter plasmid, a 2,350 bp fragment containing the *Pax6 E60* enhancer was amplified by PCR from the *E60* hsp68-LacZ-pA construct (McBride et al., 2011) using primers 5′-TGGCCTAACTGGCCGGTACCTGCAATGCTAGGGATCAAACC-3′ and 5′-TCCTCGAGGCTAGCGAGCTCATCTTGCCGGTCACCTCACTA-3′ and cloned into the NotI and SpeI restriction sites of the pGL4.23-based luciferase reporter plasmid containing a 32 bp thymidine kinase (tk) minimal promoter. The pGL4.23-Gsx1enh-tk-luc reporter was generated by amplifying by PCR a genomic fragment with primers 5′-TGCAATGCTAGG GATCAAACC-3′ and 5′-TCCTCGAGGCTAGCGAGCTCTCTTGCCGGTCACCTCA CTA-3′ from a Bac-Pac containing the Gsx1 locus (CHORI, RP24-245J15) and cloning it into the KpnI and SacI sites of the pGL4.23 vector. The 5XUAS-tk-luc reporter plasmid contains five GAL4 binding sites inserted upstream of a herpes simplex virus (HSV) thymidine kinase (tk) promoter driving the expression of the firefly luciferase. The mouse *Pax6* expression plasmid was generated by amplifying by PCR the *Pax6* ORF from a pEFX-Pax6 expression vector (obtained from E.A. Grove) using primers 5′-AAGAGGACTTGAATTCGCAGAACAGTCACAGCGGAGTG-3′ and 5′-GTTCTAGAGGCTCCAGTTACTGTAATCGAGGCCAGTAC-3′ containing EcoRI and XhoI and cloning it into the corresponding sites of a pCS2-Myc expression vector. The pCS2-Flag*-mDmrta2* has been generated by cloning the mDmrta2 ORF into the EcoRI and XhoI sites of the pCS2-Flag vector. The pCS2 Flag mDmrta2ΔDM with the DM domain deleted contains amino acids 1–62 and 125–531 of WT mDmrta2. The *mDmrta2 C-R* mutant was generated by amplifying two fragments encoding the N-terminal and C-terminal region of *Dmrta2* using primers 5′-CATTCTGCCTGGGGACGTCGGAGC-3′ and 5′-CTTCTCCGCTGCCCTCAACAGCAG-3′ for the N-terminal region, and primers 5′-GCGCGCGAGTTGCAATTGCT-3′ and 5′-CCGGGCCCAATGCATTGGCG-3′ for the C-terminal region, and assembling them with a third overlapping fragment generated in vitro (IDT, gBlocks Gene Fragment) encoding the *Dmrta2* DM domain in which an arginine replaces each cysteine, and cloning them into the HindIII and NotI sites of the pCS2-Flag vector using the Gibson Assembly Cloning Kit (New England Biolabs, catalog #E5510S). The pCDNA3-Myc*-Zfp423* expression plasmid was described previously (Masserdotti et al., 2010). The pCMV-Gal4-Dmrta2(FL) and pCMV Gal4-Dmrta2 (126–531) fusion constructs have been generated by amplifying by PCR and cloning the corresponding Dmrta2 fragments in frame with the Gal4 DBD into the BamHI site of the pCMV-Gal4 vector (Nan et al., 1997).

For coimmunoprecipitation, the *mDmrta2 **a**DM* mutant has been generated by PCR amplifying two overlapping fragments encoding the N-terminal and C-terminal region of *Dmrta2* using primers 5′-CATTCTGCCTGGGGACGTCGGAGC-3′ and 5′-CGTACAGCAATTGCAACTCGCGCGCCTTCTCCGCTGCCCTCAACAGC-3′ for the N-terminal region and primers 5′-GCGCGCGAGTTGCAATTGCT-3′ and 5′-CCGGGCCCAATGCATTGGCG-3′ for the C-terminal region and cloning them into the HindIII and NotI sites of the pCS2-Flag vector using the Gibson Assembly Cloning Kit (New England Biolabs, catalog #E5510S).

For GST pull-down assays, cDNAs encoding mDmrta2 and a deletion mutant containing only the DM domain (GST-Dmrta2 DM, containing aa 42–133) and mDmrt3 were inserted in pGEX plasmids.

### Cell culture

Embryonal carcinoma P19 cells and human embryonic kidney 293T (HEK293T) were grown in D-MEM medium (Invitrogen, catalog #61965-059) supplemented with 10% fetal bovine serum (Invitrogen, catalog #26140079) and 100 U/ml penicillin-streptomycin (Invitrogen, catalog #15140-122) and maintained in culture flasks at 37°C under 5% CO_2_. The cells were subcultured when they reached 80% confluency.

### Luciferase reporter assays

Reporter assays using the *Pax6 E60* enhancer tk-luc reporter plasmid (Pax6 E60-tk-luc) have been performed in P19 cells. Cells were plated in 12-well plates and after 24 h, transfected using the CalPhos Mammalian Transfection Kit (Takara, catalog #631312) with 500 ng of the Pax6-E60-tk-luc luciferase reporter, or an “empty” tk-luc reporter vector, with or without pCS2-Flag*-mDmrta2* and pCDNA3-Myc*-Zfp423* expression plasmids, together with a plasmid encoding the *Renilla* luciferase gene. After 48 h, cells were washed with 1× PBS, total cellular extracts were prepared, and luciferase activity was measured using the Dual-luciferase Reporter Assay System (Promega, catalog #E1960). Ratios of *luc/Renilla* luminescence were calculated and presented as fold activation for each reporter. The HDAC class I inhibitor Romidepsin (MedChemExpress, catalog #HY-15149) was used at 0.005–0.01 μm. It was added 24 h after transfection and 24 h before performing the luciferase reporter assays. At least three independent transfections have been performed for each experiments. Each dot represents the mean value obtained from one transfection performed in triplicate.

Reporter assays using the 5XUAS-tk-luc reporter have been performed in HEK293T cells. HEK293T cells (2.5 × 10^5^ cells per well) were cultured and transiently transfected as described for P19 cells with 200 ng of 5XUAS-tk-luc reporter construct, with or without pCMV-Gal4-Dmrta2, pCMV-Gal4-Dmrta2(126–531), and/or pCDNA3-Myc*-Zfp423* expression plasmids. To maintain the same amount of transfected DNA and to avoid squelching artifacts, the different amounts of cotransfected plasmid were complemented in equimolar manner by using the corresponding empty plasmid. Forty-eight hours posttransfection, cells were lysed, and luciferase activities were measured using the Single Glo luciferase reporter assay (Promega). Results were normalized for transfection efficiency using total protein content.

### GST pull-down

pCDNA3-Myc*-Zfp423* plasmid has been transcribed and translated using in vitro T7-coupled reticulocyte lysate. GST fusion proteins were incubated with labeled proteins in 200 μl binding buffer (Tris 20 mM, pH 7.4, NaCl 0.1 M, EDTA 1 mM, glycerol 10%, Igepal 0.25% with 1 mM DTT, and 1% milk) for 2 h at RT. After washing, bound proteins were separated by SDS-PAGE and visualized by Western blot.

### Coimmunoprecipitation and western blot

For coimmunoprecipitation (Co-IP), transfected HEK293T cells were lysed in IPH buffer (20 mM Tris-HCl, pH 7.5, 150 mM NaCl, 2 mM EDTA, 1% NP-40) supplemented with protease inhibitor cocktail (Roche, cOmplete EDTA-free Protease Inhibitor Cocktail, catalog #05892791001) and incubated for 15 min, at 4°C. Cell lysates were then centrifuged twice at 13,000 rpm for 30 min, at 4°C to remove debris. Part of the lysate (2.5%) was kept as a positive input control. Co-IPs were performed by incubating cell lysates (1 mg) with 5 μl of the indicated antibodies at 4°C with rotation, O/N. Then, 20 μl of magnetic beads G (Cell Signaling, catalog #9006s) was then added for 4 h, at 4°C. After four washes with IPH buffer, bound protein complexes were eluted by incubating beads in Laemmli sample buffer for 10 min, at 90°C. Immunoprecipitated protein complexes were subjected to Western blot analysis as described below.

For Western blot analysis, protein extracts from dissected cortices were prepared in RIPA buffer (50 mM Tris-HCl, pH 8, 150 mM NaCl, 1% NP-40, 0.5% sodium deoxycholate, 0.1% SDS), and those from cultured cells were prepared in IPH buffer supplemented with protease inhibitor cocktail. Homogenates were quantified by using the Bradford Assay (OD 595 nm) and centrifuged for 15 min, at 10,000 × *g*, at 4°C, and the supernatants were collected. Equal quantities of protein (60 μg/lane) were separated by 8–10% SDS-PAGE in a chamber filled with running buffer (0.1% SDS, 25 mM Tris base, 190 mM glycine; adjust pH to 8.3) and placed into a transfer cassette with transfer buffer (25 mM Tris-base, 190 mM glycine, 20% MetOH, adjust pH to 8.3), and the proteins were transferred onto a polyvinylidene difluoride (PVDF, Cytiva, catalog #10600029) membrane. Then the membrane was blocked with Tris-buffered saline (TBS) with 5% non-fat milk for 1 h, at RT, and incubated at 4°C, O/N, with the following primary antibodies: rabbit anti-Flag (1:1,000); rabbit anti-HA (1:1,000); rabbit anti-H3 (1:50,000, Millipore, catalog #07-690); mouse anti-Myc (1:1,000, Sigma, catalog #M4439); mouse anti-Zfp423 (1:1,000); and the NurD complex antibody kit (Cell Signaling, catalog #8349T). Following rinsing three times with TBS/Tween 20, the membranes were incubated with the following secondary antibodies: anti-mouse IgG HRP (1:5,000, Jackson ImmunoResearch, catalog #115-035-062) and anti-rabbit IgG HRP (1:3,000, Cell Signaling, catalog #7074) for 1 h, at RT. Protein levels were normalized to H3, and all proteins were detected using the Novex ECL Chemiluminescent Substrate Reagent Kit (Invitrogen, catalog #WP20005).

### Electrophoretic mobility shift assays

Electrophoretic mobility shift assays (EMSAs) were performed using nuclear extracts prepared from HEK293T cells transfected with expression vectors of either DMRTA2 or DMRTA2^R116P^ as described (Dignam et al., 1983). Total protein concentrations were determined by Bradford assays (Bio-Rad). DMRTA2 WT and DMRTA2^R116P^ proteins were also produced using the Transcription and Translation (TnT) system (Promega). EMSAs were performed as described previously (Van Lint et al., 1994). Briefly, 2 μl of TNT preparation or 1 μg nuclear extracts from HEK293T cells either nontransfected or transfected with DMRTA2 WT or DMRTA2 mutated were first incubated for 10 min in the absence of a probe in a reaction mixture containing 10 μg of DNase-free BSA (Bioké), 2 μg of poly(dI–dC) (Sigma) as nonspecific competitor DNA, 50 μM ZnCl_2_, 0.25 mM DTT, 20 mM HEPES, pH 7.3, 60 mM KCl, 1 mM MgCl_2_, 0.1 mM EDTA, and 10% (v/v) glycerol. A total of 30,000 cpm of probe (10–40 fmol) was then added to the mixture that was incubated for 20 min. Samples were subjected to electrophoresis at room temperature on 6% polyacrylamide gels at 120 V for 2–3 h in 1× TGE buffer (25 mM Tris-acetate, pH 8.3, 190 mM glycine, and 1 mM EDTA). Gels were dried and autoradiographed for 48–72 h at −80°C. The probe used are oligonucleotides 5RE described in Murphy et al. (2007).

### Rapid immunoprecipitation mass spectrometry of endogenous protein

Rapid immunoprecipitation mass spectrometry of endogenous protein (RIME) experiments were carried out as previously described (Mohammed et al., 2016) with minor modifications. Briefly, E12.5 cortices were dissected on ice in RNAase-free ice-cold 1× PBS and fixed for 15 min in 1% formaldehyde (Thermo Scientific, catalog #28906) in 1× PBS at RT. The cross-linking was quenched by adding glycine to a final concentration of 125 mM and incubating samples for 8 min. Subsequently, the fixed tissues were pelleted and washed in ice-cold PBS. Nuclear extraction was done using ice-cold LB1 buffer [50 mM HEPES-KOH, pH 7.5, 140 mM NaCl, 1 mM EDTA, 10% glycerol, 0.5% NP-40 (vol/vol) and 0.25% Triton X-100 (vol/vol)], incubating cells on rotation for 10 min at 4°C. The suspension was then centrifuged at 2,000 × *g* for 5 min at 4°C and the obtained nuclear pellet was washed in ice-cold LB2 buffer (10 mM Tris-HCl, pH 8.0, 200 mM NaCl, 1 mM EDTA, 0.5 mM EGTA). Pelleted nuclei were subsequently resuspended in LB3 buffer [10 mM Tris-HCl, pH 8.0, 100 mM NaCl, 1 mM EDTA, 0.5 mM EGTA, 0.1% sodium deoxycholate (wt/vol) and 0.5% N-lauroylsarcosine (wt/vol)] and, after an incubation of 15 min, were subjected to three cycles of sonication (30 s ON/30 s OFF for 10 min, at 200 W, high setting, Bioruptor, Diagenode). Triton X-100 was added to the sonicated lysate to a final concentration of 1%. The obtained chromatin was then cleared by centrifugation at 20,000 × *g* for 10 min, at 4°C, and the supernatant was collected. All lysis buffers were supplemented with protease inhibitors before use.

Antibody-bound beads were generated by incubating 100 μl of Protein G magnetic beads (Cell Signaling, catalog #9906) with 5 μl rabbit anti-HA (Abcam, catalog #ab9110) or rabbit anti-IgG (Merck, catalog #12-370) in PBS/BSA and incubated in rotation at 4°C, O/N. The next day, the antibody-bound beads were washed three times in PBS/BSA to remove any unbound antibodies. For immunoprecipitation, 2 mg of protein lysate was incubated O/N, with 100 µl antibody-bound beads at 4°C. The next day, the bead-bound complexes were washed nine times in RIPA buffer (50 mM HEPES, pH 7.6, 1 mM EDTA, 0.7% sodium deoxycholate, 1% NP-40, and 0.5 M LiCl) at 4°C and then washed two times with a cold 100 mM ammonium hydrogen carbonate (AMBIC) solution. Finally, the beads were transferred to new tubes, snap-frozen at −80°C, and stored at this temperature until mass spectrometry analysis.

### Mass spectrometry

The samples were treated using filter-aided sample preparation (FASP). To first wash the filter, 100 µl of 1% formic acid was placed in each Millipore Microcon 30 MRCFOR030 Ultracel PL-30, and samples were centrifuged for 15 min, at 14,500 rpm. For protein adjustment, 40 µg of protein in 150 µl of 8 M urea buffer (urea 8 M in 0.1 M Tris, pH 8.5) was placed individually in a column and centrifuged for 15 min, at 14,500 rpm. The filtrate was discarded, and the columns were washed three times with 200 µl of urea buffer followed by centrifugation for 15 min, at 14,500 rpm. For the reduction step, 100 µl of dithiothreitol (DTT) was added and mixed for 1 min, at 400 rpm with a thermomixer; incubated for 15 min, at 24°C; and centrifuged for 15 min, at 14,500 rpm. The filtrate was discarded and 100 µl of urea buffer was added before another centrifugation for 15 min, at 14,500 rpm. An alkylation step was performed by adding 100 µl of iodoacetamide (IAA), in urea buffer in the column, mixing for 1 min, at 400 rpm, incubating for 20 min in the dark, and centrifuging for 10 min, at 14,500 rpm. To remove the excess IAA, 100 µl of urea buffer was added and the samples were centrifuged for 15 min, at 14,500 rpm. To quench the remaining IAA, 100 µl of DTT was added to the column; mixed for 1 min, at 400 rpm; incubated for 15 min, at 24°C; and centrifuged for 10 min, at 14,500 rpm. Excess DTT was removed by adding 100 µl of urea buffer and centrifuging for 15 min, at 14,500 rpm. The filtrate was discarded, and the column was washed three times with 100 µl of sodium bicarbonate buffer 50 mM (ABC) followed by centrifugation for 10 min, at 14,500 rpm. The last 100 µl were kept at the bottom of the column to avoid any evaporation in the column. For digestion, 80 µl of mass spectrometry grade trypsin (1/50 in ABC buffer) was added to the column; mixed for 1 min, at 400 rpm; and incubated at 24°C, O/N in a water-saturated environment. The Microcon columns were placed on a LoBind tube of 1.5 ml and centrifuged for 10 min, at 14,500 rpm. Then, 40 µl of ABC buffer was added to the column before centrifugation for 10 min, at 14,500 rpm. Then, 10% trifluoroacetic acid (TFA) was added to the content of the LoBind tube to obtain 0.2% TFA. The samples were dried in a SpeedVac up to 20 µl and transferred for injection.

The samples were analyzed using nano-LC-ESI-MS/MS (timsTOF Pro, Bruker) coupled with a UHPLC nanoElute (Bruker). Liquid chromatography was separated at 50°C and at a flow rate of 200 nl/min by nanoUHPLC (nanoElute, Bruker) on a C18 column (25 cm × 75 μm ID) with integrated CaptiveSpray insert (Aurora, IonOpticks). Mobile phases A and B were water with 0.1% formic acid (v/v) and ACN with formic acid 0.1% (v/v), respectively. Samples were loaded directly on the analytical column at a constant pressure of 800 bar. The digest (1 µl) was injected, and the organic content of the mobile phase was increased linearly from 2 to 15% B within 40 min, followed by an increase to 25% B within 15 min, and further to 37% B in 10 min, followed by a washing step at to 95% B in 5 min. Data acquisition on the timsTOF Pro was performed using Hystar 6.1 and tims Control 2.0. The timsTOF Pro data were acquired using 160 ms TIMS accumulation time, and mobility coefficients (1/K0) range from 0.75 to 1.42 Vs/cm^2^. Mass spectrometry analysis was carried out using the parallel accumulation-serial fragmentation (PASEF; Meier et al., 2018) acquisition method. One MS spectra followed by PASEF MSMS spectra per total cycle of 1.16 s. All MS/MS samples were analyzed using Mascot (Matrix Science; version 2.8.1). Scaffold (version Scaffold_5.0.0, Proteome Software) was used to validate MS/MS-based peptide and protein identifications. The mass spectrometry proteomics data have been deposited to the ProteomeXchange Consortium via the PRIDE (Perez-Riverol et al., 2025) partner repository with the dataset identifier PXD062221 and 10.6019/PXD062221.

### Human subjects and clinical evaluation

As a result from a GeneMatcher match (Sobreira et al., 2015), we studied a Pakistani consanguineous family with three affected individuals, V-3, V-5, and V-7 (Fig. 6*A*,*C*). The clinical evaluation of the probands was performed at the Aga Khan University Hospital, Karachi. Detailed clinical evaluation was carried out in two affected persons, V-3 and V-5 (Extended Data Table 6-1); however, the affected person V-7 showed similar clinical features. This study was conducted with ethical approval from the Aga Khan University Hospital, Karachi. We obtained written informed consent, and blood samples were taken from all the available persons (III-7, IV-1, IV-5, IV-7, V-3, V-5, V-7) in the family. Genomic DNA was extracted as described previously (Sambrook and Russell, 2006). The MRI of V-3 (6 years) was performed, which revealed microcephaly, a simplified gyral pattern along with agenesis of the corpus callosum and colpocephaly. Electroencephalography (EEG) was performed on the affected person, V-5 (5 years).

### Whole-exome sequencing

We performed whole-exome sequencing (WES) using DNA samples of two affected individuals, V-3 and V-5 (Fig. 6*A*,*C*). WES and further data analysis to find the exact causative variant has been performed as described (Yousaf et al., 2022, 2023). The combined analysis of WES data of V-3 and V-5 revealed three shared homozygous variants: *C1orf109*: NM_001303030.2: c.373C > A:p. (Leu125Ile), *DMRTA2*: NM_032110.3: c.347G > C:p.(Arg116Pro), and *TMEM161A*: NM_001256766.3: c.745G > A:p.(Ala249Thr) between both the WES-analyzed persons. We further excluded two variants located in *C1orf109* and *TMEM161A* genes, as the variant in *C1orf109* was found 11× in homozygous state in the gnomAD database (v4.1.0), and the *TMEM161A* variant did not segregate with the phenotype. *DMRTA2*: p. (Arg116Pro) is the only homozygous variant that segregated (tested by Sanger sequencing) with the phenotype in this consanguineous family (Fig. 6). The variant p. (Arg116Pro) has a Combined Annotation Dependent Depletion (CADD) score (v1.6) of 31 (Kircher et al., 2014) and is predicted as “deleterious” by multiple in silico-prediction programs and classified as a variant of uncertain significance, according to the ACMG classification criteria PM2, PP1, PP3, and PP4 (Richards et al., 2015).

### Statistical analysis

Statistical significance was determined by the Student's *t* test or the one-way analysis of variance (ANOVA), with a threshold for significance set to *p* < 0.05. All results are plotted as the mean ± SD, as indicated in the figure legends.

## Results

### *Dmrta2* cooperates with *Pax6* in defining telencephalic dorsoventral compartments and acts through its repression to control cortical patterning

**Figure 1.**
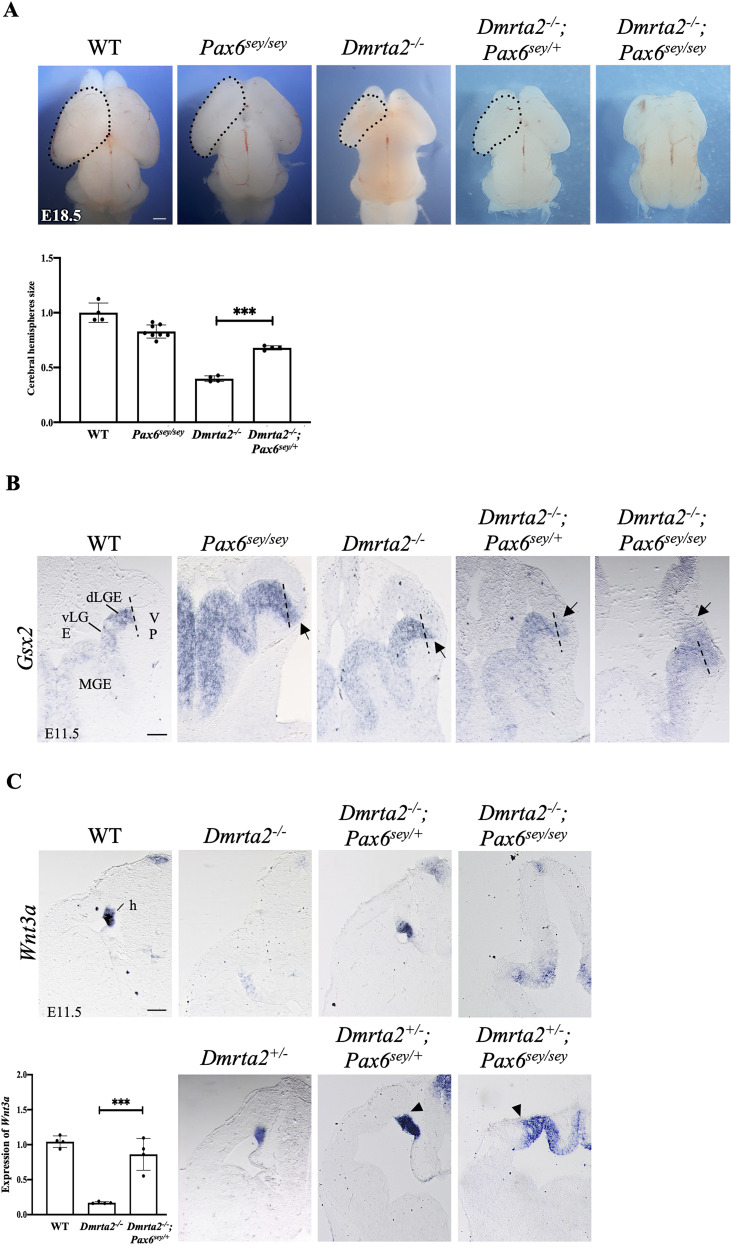
Cortical phenotype of *Dmrta2^−/−^* embryos in the absence of one or two Pax6 alleles. ***A***, Dorsal views of the brain of E18.5 embryos of the indicated genotypes. Scale bar, 100 μm. Graphs representing the measured surface area of the cerebral hemispheres of *Pax6^sey/sey^*, *Dmrta2^−/−^* and *Dmrta2^−/−^; Pax6^sey/+^* compared with WT set to 1 are shown. ***p < 0.001, Student's *t* test. Note that the reduction in cerebral hemisphere size observed in *Dmrta2^−/−^* embryos is less severe in the absence of a Pax6 allele. An analysis of the size of the cerebral hemispheres in these embryos collected at E11.5 and E15.5 embryos is presented in Extended Data Figure 1-1. ***B***, Coronal sections through the brain of E11.5 embryos of the indicated genotypes processed by ISH for *Gsx2*. Note that *Gsx2* expression in the telencephalon only slightly crosses the pallium-subpallium (PSB) boundary in *Pax6^sey/sey^*, *Dmrta2^−/−^* and *Dmrta2^−/−^; Pax6^sey/+^*embryos, and robustly expands into the telencephalon of *Dmrta2^−/−^; Pax6^sey/sey^* embryos. The PSB region is indicated by a dashed line. Arrows point to the limit of the expansion of *Gsx2* expression within the dorsal telencephalon. Scale bar, 500 μm. An analysis of *Gsx1* expression in embryos of the same genotype is presented in Extended Data Figure 1-2. ***C***, Coronal sections through the brain of E11.5 embryos of the indicated genotype processed by ISH for the expression of *Wnt3a* marking the hem. Arrowheads indicate the dorsal extent of *Wnt3a* expression in the medial pallium detected in *Dmrta2^+/−^; Pax6^sey/+^* and *Dmrta^+/−^; Pax6^sey/sey^*. h, hem. Scale bar, 500 μm. Quantitative RT-qPCR analysis of *Wnt3a* in dissected cortices of *Dmrta2^−/−^*, *Dmrta2^−/−^; Pax6^sey/+^*, and WT control embryos is shown on the left. Results are normalized to the level of the expression detected in the cortex of WT embryos. ***p < 0.001, Student's *t* test. Note that the reduction of *Pax6* partially rescues cortical hem formation in *Dmrta2* homo- and heterozygous mutant embryos. A similar analysis of *Wnt8b* expression in *Dmrta2^+/−^* embryos and in *Dmrta2^+/−^* embryos with one or two *Pax6^sey^* alleles is presented in Extended Data Figure 1-3.

To test the hypothesis that *Dmrta2* acts through *Pax6* to control cortex development, we generated double mutants by intercrossing *Dmrta2^+/−^* mice with *Pax6^sey/+^* mice which have a point mutation in the *Pax6* gene creating a null allele (Hill et al., 1991). Embryos were collected at three embryonic time points (E11.5, E15.5, and E18.5) to measure the surface area of their hemispheres. We found that the cerebral hemispheres of *Pax6^sey/sey^* embryos and *Dmrta2^−/−^* embryos were smaller compared with embryos at E18.5, with the reduction in *Dmrta2^−/−^* being more severe (−60.1% ± 2.5% in *Dmrta2^−/−^* compared with −16.9% ± 7.2% in *Pax6^sey/sey^*) and detected at an earlier stage in *Dmrta2^−/−^* embryos than in *Pax6^sey/sey^* embryos. In the *Dmrta2^−/−^; Pax6^sey/sey^* embryos, the cerebral hemispheres were virtually absent. Notably, the extent of the reduction in *Dmrta2^−/−^* embryos was less severe in the absence of one allele of *Pax6* (−31.6% ± 6.5% in *Dmrta2^−/−^; Pax6^sey/+^* compared with −60.1% ± 2.5% in *Dmrta2^−/−^*; Fig. 1*A*). This rescue was already visible at earlier stages: at E11.5 (−38.6% ± 5.6% in *Dmrta2^−/−^; Pax6^sey/+^* compared with −48.2% ± 6.4% in *Dmrta2^−/−^*) and E15.5 (−34.5% ± 4.2% in *Dmrta2^−/−^; Pax6^sey/+^* compared with −48.9% ± 7.7% in *Dmrta2^−/−^*; Extended Data Fig. 1-1). Analysis by in situ hybridization of the expression of the subpallial marker *Gsx2* expressed at a high level in progenitors of the dorsal LGE (dLGE) revealed that its expression in the single KO embryos and in *Dmrta2^−/−^; Pax6^sey/+^ mutants* remained confined to the subpallium or only very slightly crossed the pallium-subpallium boundary (PSB) while it distinctly extended into the abortive cortical primordium of the double KO embryos (Fig. 1*B*). Similarly, *Gsx1* that is also bound by Dmrta2 (Konno et al., 2019) and is expressed mainly in MGE and vLGE (Pei et al., 2011) remains restricted to the ventral telencephalon in *Pax6^sey/sey^*, *Dmrta2^−/−^*, and *Dmrta2^−/−^; Pax6^sey/+^*embryos but appears to expand into the reduced telencephalon of *Dmrta2^−/−^; Pax6^sey/sey^* embryos (Extended Data Fig. 1-2). These data provide additional evidence for the importance of a correct balance between *Dmrta2* and *Pax6* for normal cortical hemisphere growth. They also reveal that *Dmrta2* and *Pax6* cooperate in maintaining cortical identity in dorsal telencephalic progenitors.

10.1523/ENEURO.0377-24.2025.f1-1 Figure 1-1 **The growth of the telencephalic vesicles is rescued upon the loss of one *Pax6* allele in *Dmrta2*^-/-^ embryos. (A)** Dorsal views of the brain of E11.5 and E15.5 embryos of the indicated genotype, Scale bar, 100μm. **(B)** Graphs representing the surface area of E11.5 and E15.5 cerebral hemispheres compared to WT set to 1.*P < 0.05, **P < 0.01, Student’s *t*-test. Download Figure 1-1, TIF file.

**Figure 2.**
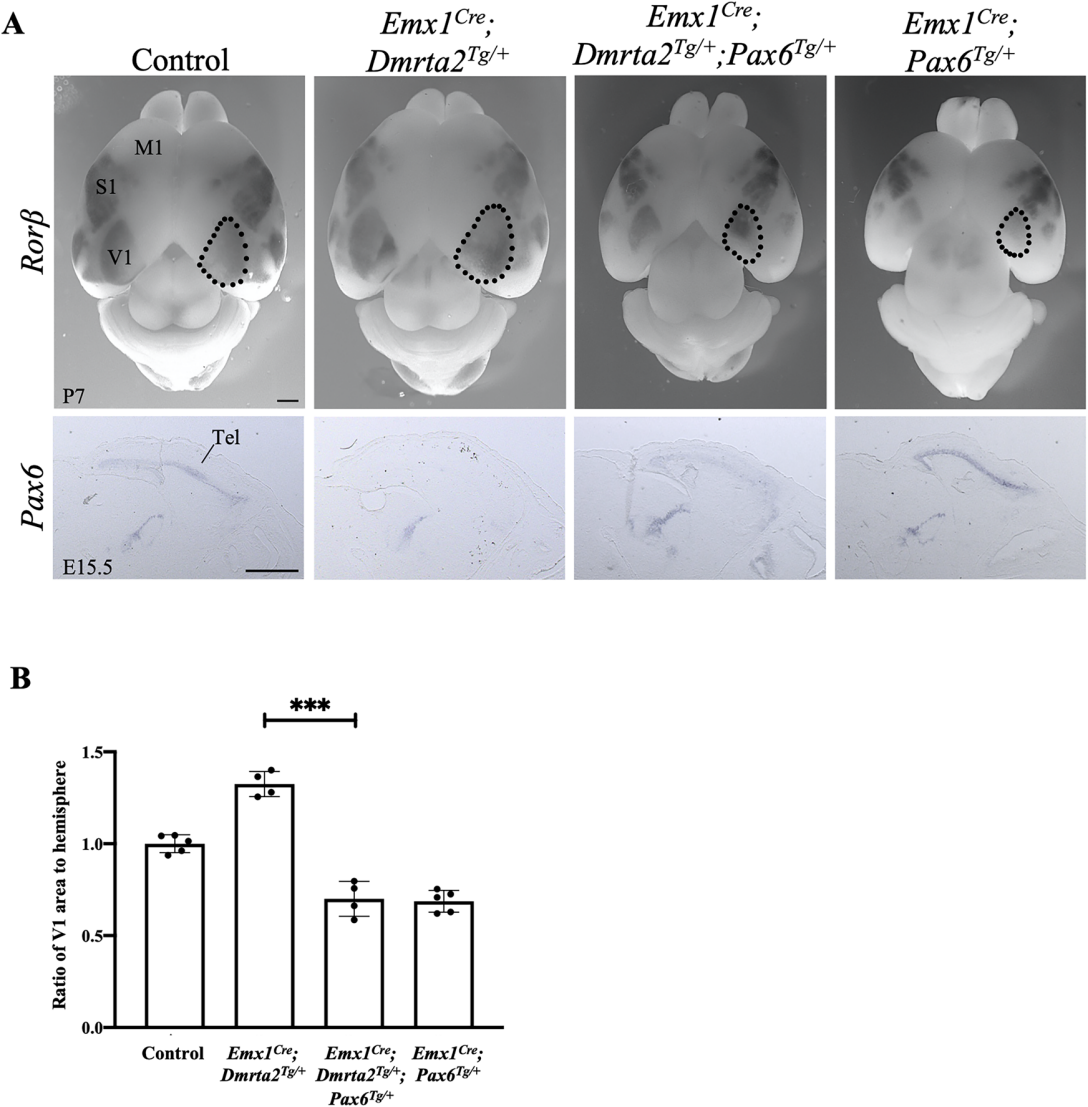
*Pax6* overexpression rescues the expansion of the V1 area observed in *Dmrta2* overexpressing transgenics. ***A***, Dorsal views of the brain of P7 neonates of the indicated genotype processed by whole-mount ISH for *Rorβ* expression. *Dmrta2^Tg/+^; Pax6^Tg/+^* were used as controls. Sagittal sections of the brain of E15.5 embryos of the corresponding genotype with *Pax6* expression detected by ISH are shown below. Tel, telencephalon; M1, primary motor area; S1, primary somatosensory area; V1, primary visual areas. Scale bar, 100 μm. ***B***, Histograms show that as a ratio of surface area to total hemisphere size, V1 expansion is significantly rescued by the reduction of *Pax6*. ***p < 0.001, Student's *t* test.

10.1523/ENEURO.0377-24.2025.f1-2 Figure 1-2 **The ventral determinant *Gsx1* expands dorsally in the abortive cortical primordium of *Dmrta2^-/-^; Pax6^Sey/Sey^* embryos.** Coronal brain sections through the brain of E11.5 embryos of the indicated genotypes processed by ISH for *Gsx1*. Note that *Gsx1* expression remains restricted to the ventral telencephalon in *Pax6^sey/sey^*, *Dmrta2^-/-^* and *Dmrta2^-/-^; Pax6^sey/+^* embryos but appears to expand dorsally into the telencephalon in *Dmrta2^-/-^; Pax6^sey/sey^* embryos. Arrows point to the dorsal limit of *Gsx1* expression. The pallium-subpallium (PSB) boundary region is indicated by a dashed line. Abbreviations: MGE, medial ganglionic eminence; PSB, pallium/subpallium boundary; vLGE, ventral lateral ganglionic eminence; VP, ventral pallium. Download Figure 1-2, TIF file.

**Figure 3.**
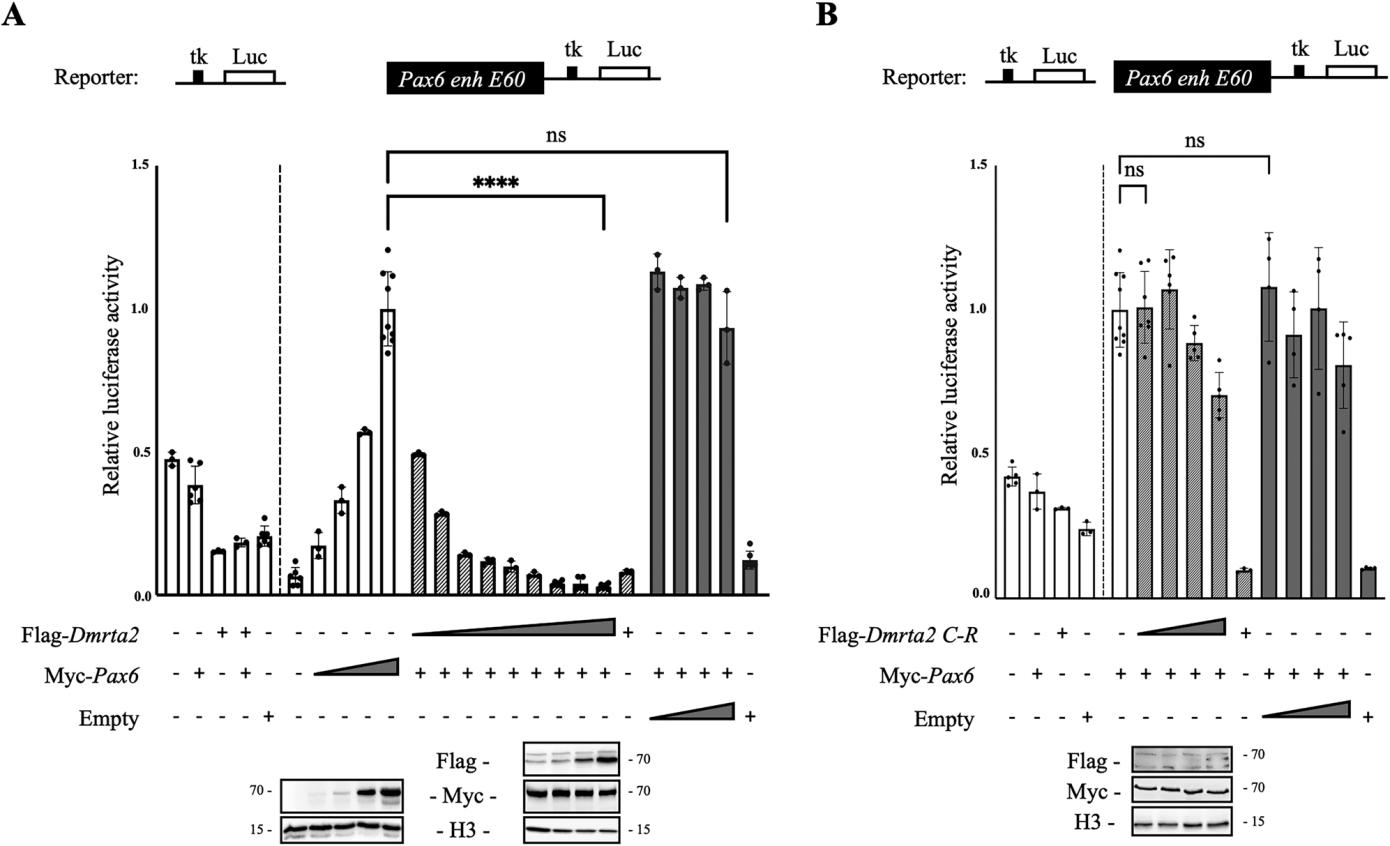
*Dmrta2* inhibits the activation by *Pax6* of the *Pax6 E60* enhancer. In accordance with *Pax6* identification as a direct Dmrta2 target, results presented in Extended Data Figure 5-1 show that Dmrta2 regulates *Pax6* in a Wnt-independent manner. ***A***, Reporter assays in P19 cells transfected with a *Pax6 E60* tk-luc reporter vector, or an “empty” tk-luc reporter vector as indicated, together with increasing doses (from 62.5 to 500 ng) of a Myc*-Pax6* expression vector and/or increasing doses (from 2 to 500 ng) of a Flag*-Dmrta2* expression vector at as indicated. Note that the *Pax6* dose-dependent transactivation of the *E60* tk-luc reporter vector is attenuated by *Dmrta2*. Western blots showing the expression levels of Myc*-Pax6* and Flag*-*Dmrta2 (from 62.5 to 500 ng) are shown below. *n* = 3. NS, not significant. ***p < 0.0001, one-way ANOVA test. ***B***, Reporter assays in P19 cells transfected with a *Pax6* E60 tk-luciferase reporter vector or an “empty” tk-luc reporter vector as indicated, with a Myc*-Pax6* expression vector and/or a Flag*-Dmrta2* point mutant with the cysteine residues of the zinc finger motif replaced by arginine residues (Flag*-Dmrta2 C-R*) at increasing doses, as indicated. Western blots showing the expression levels of Myc*-Pax6* and Flag*-Dmrta2 C-R* are shown below. Note that in contrast to the WT protein, the Flag*-Dmrta2 C-*R is unable to repress *Pax6* transactivation of the *E60* reporter construct. Extended Data Figure 3-2 shows that as the WT protein, the Flag*-Dmrta2 C-*R mutant protein localizes to the nucleus. Reporter assays showing that Dmrta2 also represses the activity of an enhancer identified in the *Gsx1* locus are presented in Extended Data Figure 3-3.

10.1523/ENEURO.0377-24.2025.f1-3 Figure 1-3 **Reduction of *Pax6* partially rescues medial cortical fate in *Dmrta2* homo- and heterozygous mutant embryos. (A)** Coronal sections through the brain of E11.5 embryos of the indicated genotype processed by ISH for the expression of *Wnt8b*** **marking the dorsomedial telencephalic primordium. Arrowheads indicate the dorsal extent of *Wnt8b* expression detected in the pallium. The extent of the hem as revealed by more intense *Wnt8b* expression in *Dmrta2^+/-^* and in *Dmrta2^+/-^*;* Pax6^sey/+^* is indicated by brackets. Et: eminentia thalami; h, hem. Scale bar, 500 μm.** (B) **Quantitative RT–qPCR analysis of *Wnt8b* in dissected cortices of *Dmrta2^-/-^*, *Dmrta2^-/-^*;* Pax6^sey/+^* and WT control embryos is shown on the left. Results are normalized to the level of the expression detected in the cortex of WT embryos. ***p < 0.001, Student’s *t*-test. Download Figure 1-3, TIF file.

*Pax6* suppresses medial cortical fate in the cortical neuroepithelium (Godbole et al., 2017). Therefore, and because *Pax6* is excluded from the hem (Saulnier et al., 2013; De Clercq et al., 2018), we hypothesized that the medial expansion of *Pax6* observed upon the loss of *Dmrta2* could be responsible for the suppression of hem formation. To test this hypothesis, we examined by in situ hybridization the expression of *Wnt3a* which identifies the cortical hem, and *Wnt8b*, which marks the dorsomedial telencephalic primordium and eminentia thalami, both being upregulated in the absence of *Pax6* (Godbole et al., 2017), in the cortex of E11.5 homozygous and heterozygous *Dmrta2* mutants with the loss of one *Pax6* allele.

As previously reported (Saulnier et al., 2013), in the medial telencephalon of *Dmrta2^−/−^* embryos, *Wnt3a* is strongly reduced. As shown in Figure 1*C*, *Wnt3a* is much less affected in *Dmrta2^−/−^; Pax6^sey/+^* embryos than in *Dmrta2^−/−^* embryos. RT-qPCR on RNA extracted from telencephalic tissue isolated from E11.5 embryos confirmed the drastic downregulation of *Wnt3a* in *Dmrta2^−/−^* embryos and that a rescue of its expression occurs upon the loss of one *Pax6* allele. While compared with controls *Wnt3a* is slightly reduced in intensity in *Dmrta2^+/−^* embryos, its expression domain is expanded in *Dmrta2^+/−^; Pax6^sey/+^* embryos and even more in *Dmrta2^+/−^; Pax6^sey/sey^* embryos. Such an expansion is however not visible anymore in the abortive cortex of the *Dmrta2^−/−^; Pax6^sey/sey^* embryos.

As reported previously, in the medial telencephalon of *Dmrta2^−/−^* embryos, *Wnt8b* is downregulated in the telencephalon but not in the eminentia thalami (Saulnier et al., 2013). In *Dmrta2^+/−^* embryos, such a phenotype is not observed and *Wnt8b* appears similar to controls. In *Dmrta2^+/−^; Pax6^sey/+^* embryos, *Wnt8b* expression domain appears however to extend more into the lateral part of the telencephalon (1.2 X ± 0.06, measuring *Wnt8b* expression domain from the ventral extent of the telencephalon) than in *Dmrta2^+/−^* embryos. The hem region itself as suggested by the more intense *Wnt8b* expression appears also extended in these *Dmrta2^+/−^; Pax6^sey/+^* embryos, and even more in *Dmrta2^+/−^; Pax6^sey/sey^* embryos, as observed for *Wnt3a* (Extended Data Fig. 1-3). Thus, mediolateral patterning of the cerebral cortex and cortical hem formation is regulated by *Dmrta2* through repression of *Pax6*.

*Pax6* promotes rostral area identity in the developing neocortex (Bishop et al., 2000, 2002; O’Leary and Sahara, 2008). *Pax6* is reduced in the cortex of transgenic mice conditionally overexpressing *Dmrta2* that have expanded V1 area (De Clercq et al., 2018). It could therefore be that it is the decreased level of *Pax6* that caudalizes the neocortex of mice expressing in excess *Dmrta2*. Therefore, we generated transgenic mice conditionally overexpressing both *Dmrta2* and *Pax6* by crossing *Emx1^Cre^; Dmrta2^Tg/+^* (De Clercq et al., 2018) with *Emx1^Cre^; Pax6^Tg/+^* mice (Berger et al., 2007). We examined area formation at P7 in the cortex of heterozygous double transgenics (*Emx1^Cre^; Dmrta2^Tg/+^; Pax6^Tg/+^*) and single transgenic mice (*Emx1^Cre^; Dmrta2^Tg/+^* and *Emx1^Cre^; Pax6^Tg/+^*), as homozygous ones exhibit early lethality, possibly due to sustained expression of *Dmrta2* and *Pax6* in postmitotic cortical cells. This analysis was carried out by whole-mount in situ hybridization monitoring *Rorβ* expression, which demarcates the S1, A1, and V1 area, measuring the size of the V1 area relative to the total hemisphere size (Fig. 2). We found that the V1 area that are expanded in *Dmrta2* overexpressing mice are reduced when *Pax6* is also overexpressed, as observed in *Pax6* overexpressing mice. These findings support the hypothesis that *Dmrta2* controls cortical arealization by regulating the level of *Pax6* expression.

### Dmrta2 can act as a DNA-binding repressor on the *Pax6 E60* enhancer and a point mutation in its DM domain causes microcephaly in human

*Pax6* has been shown to be deregulated by the loss or gain of function of *Dmrta2* in the developing cortex (Konno et al., 2012; Saulnier et al., 2013). The deregulation of *Pax6* is rapid as it is already observed at E12.5 in *Nestin^Cre^; Dmrta2^fl/fl^* mice (De Clercq et al., 2018). It can be seen already at E9.5 in *Dmrta2* null mutants before the disruption of Wnt signaling that can be detected from E10.5 in Wnt reporter *BAT-gal* mice (Maretto et al., 2003; Extended Data Fig. 3-1), suggesting *Pax6* is a direct Dmrta2 target. In accordance, ChIP-seq experiments have revealed that Dmrta2 binds to the *Pax6 E60* enhancer, an ultraconserved cis-regulatory region located 25 kb downstream of *Pax6* in the large final intron of the adjacent *Elp4* gene, that contains several potential Dmrta2 binding sites and drives complex expression of *Pax6* within the nervous system, including in the telencephalon (McBride et al., 2011 ; Konno et al., 2019). To address the possibility that Dmrta2 affects *Pax6 E60* enhancer activity and investigate its mechanism of action, we performed transfection experiments in P19 mouse pluripotent embryonal carcinoma cells that have neurogenic potential (Farah et al., 2000). We have selected this cell line as it expresses at high levels early neural marker such as Sox2 but not Pax6 (Babuska et al., 2010; Leszczyński et al., 2020) nor, most likely, Dmrt5 and has been used previously for in vitro studies on enhancer activation by cortical transcription factors (Mariani et al., 2012; Pattabiraman et al., 2014 ). We transfected P19 cells with a luciferase reporter construct driven by a minimal tk promoter linked to the *Pax6 E60* enhancer. As Pax6 autoregulates its expression (Aota et al., 2003 ), we transfected either naive or *Pax6* overexpressing cells with this reporter, in the absence or presence of *Dmrta2* expression vectors. We found that while Pax6 overexpression had no significant effect on the control enhancer-less tk-luc reporter, it increased the activity of the *E60* enhancer. In the presence of Pax6, we found that Dmrta2 represses the activity of the *E60* enhancer in a dose-dependent manner (Fig. 3*A*). Such repression was not observed with cotransfection of a construct encoding a *Dmrta2* mutant (with the cysteine residues of the zinc finger motif replaced by arginine residues, designated *Dmrta2 C-R*) that without affecting its subcellular localization abolishes its ability to bind DNA (Fig. 3*B*, Extended Data Fig. 3-2). We also found that Dmrta2 also represses the enhancer activity of a tk-luc reporter construct under the control of the conserved region bound by Dmrta2 identified in the *Gsx1* locus (Konno et al., 2019; Extended Data Fig. 3-3). These findings indicated that Dmrta2 functions as a DNA-binding transcription repressor and suggested that it may attenuate *Pax6* by blocking its autoregulation.

10.1523/ENEURO.0377-24.2025.f3-1 Figure 3-1 ***Pax6* is already upregulated by the loss of *Dmrta2* at E9.5 before the Wnt signaling pathway appears to be affected.** (**A**) Whole-mount X-gal staining on *BAT*^+^/*Dmrta2*^+*/*+^ and *BAT*^+^/*Dmrta2^-/-^* embryos at E9.5 and E10.5. Note on the whole-mount views (arrowheads) and on the sections and magnification views shown on the left that the staining detected in BAT^+^/*Dmrta2^-/-^* embryos is similar to that of *BAT*^+^/*Dmrta2*^ +*/*+^ embryos at E9.5 but is reduced at E10.5. Scale bar, 200 μm. **(B)** RT–qPCR analysis of *Pax6, Emx2,* and *Lhx2* expression in the cortex of *Dmrta2^-/-^* and WT embryos. Results are normalized to the level of expression in the cortex of WT embryos. Note that *Pax6* is already upregulated in the cortex of *Dmrta2^-/-^* at E9.5, which is not the case for *Emx2* and *Lhx2,* whose deregulation is only observed from E10.5. Error bars indicate SDs of at least three independent experiments. *P <0.05 ,**P < 0.01. **(C)** Whole mount ISH analysis of *Pax6* expression shows that its upregulation (arrowheads) in *Dmrta2^-/-^* embryos can already be seen from E9.5. Scale bar, 200 μm. Download Figure 3-1, TIF file.

10.1523/ENEURO.0377-24.2025.f3-2 Figure 3-2 **The Flag-*Dmrta2 C-R* is detected in the nucleus as the WT protein in transfected P19 cells.** Flag immunostaining and DAPI staining are shown, together with a merged image. Scale bar, 10 μm. Download Figure 3-2, TIF file.

10.1523/ENEURO.0377-24.2025.f3-3 Figure 3-3 **Dmrta2 represses the activity of an enhancer identified in the *Gsx1* locus. (A)** Mouse genomic region surrounding the *Gsx1* gene (top) with ChIP-seq data from Konno et al., 2019 showing a conserved region bound by Dmrta2 and Dmrt3 approximately 16 kb downstream of *Gsx1*. **(B)** Reporter assays in P19 cells transfected with a tk-luc reporter with this conserved *Gsx1* non-coding region upstream, or an“empty ”tk-luc reporter vector as indicated, with or without increasing amount (62.5, 125, 250, and 500 ng) of *Dmrta2* or a pCS2 “empty ” plasmid. Ns: not significant, ***P < 0.0001, one-way ANOVA test. Download Figure 3-3, TIF file.

**Figure 4.**
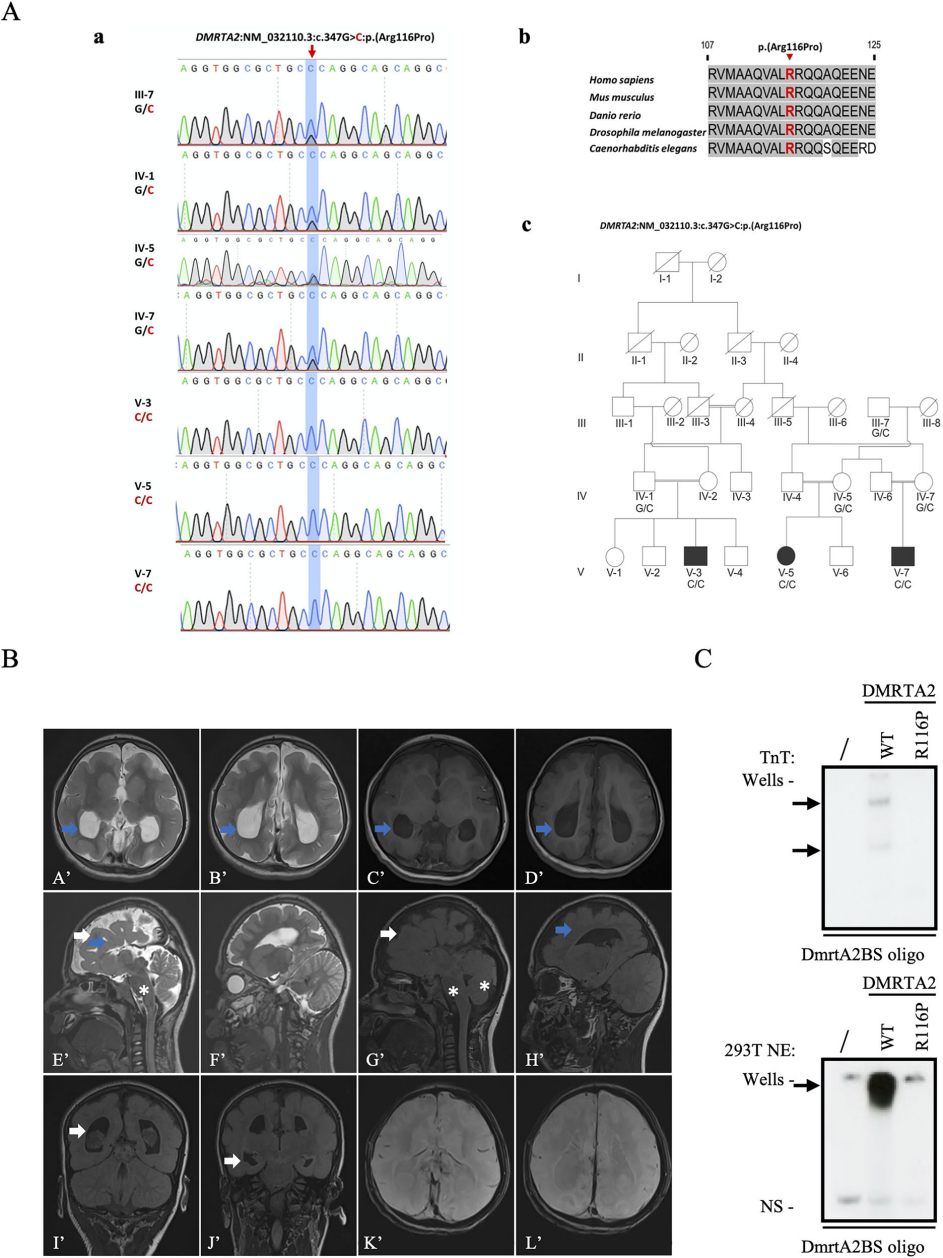
The homozygous missense variant in *DMRTA2* causes microcephaly in human. ***A***, Sanger sequencing chromatograms (***a***) showing de novo G to C heterozygous and homozygous changes in sequence causing p.Arg116Pro coding change in *DMRTA2* identified in individuals of a consanguineous family. An alignment of the sequence of the central portion of the major groove recognition helix of human, murine fish, *Drosophila*, and *C. elegans Dmrta2* is shown (***b***), together with the pedigree (***c***) of the Pakistan family in which the mutation has been identified. Symbols marked by a slash indicate that the subject is deceased. Males are indicated by squares; females are indicated by circles. Blackened symbols represented individuals who were identified as patients according to clinical examination. ***B***, Magnetic resonance (MRI) images of the brain phenotype. First row, Axial T2-weighted (***A’***, ***B’***) and T1-weighted (***C’***, ***D’***) images showing, colpocephaly, i.e., dilated posterior horns (blue arrows), with relatively parallel orientation of the bilateral lateral ventricles suggestive of complete agenesis of the corpus callosum. Second row, Sagittal T2-weighted (***E’***, ***F’***) and T1-weighted (***G’***, ***H’***) images showing pachygyria (white arrows) and absent cingulate gyrus (blue arrows). The brainstem and cerebellum are normal (white asterisks). Third row, Coronal fluid-attenuated inversion recovery (FLAIR) sequences (***I’***, ***J’***), showing enlarged occipital as well as temporal horns with no periventricular white matter changes. Axial SWI (susceptibility weighted images; ***K’***, ***L’***) does not show any calcifications. Details of the clinical characterization of the V-3 and V-5 patients are provided in Extended Data Table 4-1. ***C***, EMSA with a labeled DNA probe containing a DMRTA2 consensus binding motif (DmrtA2BS) incubated with (top panel) DMRTA2 WT or DMRTA2^R116P^ proteins produced by TnT and (bottom panel) nuclear extracts from HEK293T cells either nontransfected (/) or transfected with DMRTA2 WT or DMRTA2^R116P^. Proteins present in the binding reactions are indicated above. Arrows indicate the position of the DNA-protein complexes. “NS” indicates nonspecific complexes.

By exome sequencing, a homozygous single base pair deletion (c.1197delT) predicted to result in a frameshift variant p. (Pro400Leufs*33) in *DMRTA2* has been identified in a consanguineous family with three siblings affected by a severe prenatal neurodevelopmental disorder characterized by frontoparietal pachygyria, agenesis of the corpus callosum, and progressive severe microcephaly (Urquhart et al., 2016). To our knowledge, this is the only case report suggesting that loss of DMRTA2 causes cortical malformation in humans. Independent reporting of biallelic variants in *DMRTA2* in individuals with the same or similar phenotypes is thus required to attain the level of evidence required for a definite gene–disease relationship. Here, we identified a homozygous missense variant in the DM domain of *DMRTA2*:NM_032110.3:c.347G > C:p.(Arg116Pro) that segregated with cortical malformations in three branches of a consanguineous family, and arginine at position 116 is extremely conserved down to *Caenorhabditis elegans*, suggesting that the novel DMRTA2^R116P^ mutation is the most likely cause of the phenotype (Fig. 4*A*). Patients presented global developmental delay, dysarthria, muscle atrophy, aggressive behavior, and peripheral neuropathy and were unable to stand and walk. MRI analysis in one affected person (V-3) revealed microcephaly with agenesis of the corpus callosum, as observed in *Dmrta2^−/−^* mice (Saulnier et al., 2013). They also present symptoms of colpocephaly that is the selective dilatation of the occipital horns of the lateral ventricles, often associated with agenesis or dysgenesis of the corpus callosum (Fig. 4*B*). Electroencephalogram (EEG) was performed in V-5, which revealed abnormalities indicating a mild encephalopathy. Full details of the clinical and genetic characterization of the V-3 and V-5 patients are provided in Extended Data Table 4-1.

10.1523/ENEURO.0377-24.2025.t4-1 Table 4-1 Clinical evaluation of V-3 and V-5 patients. Download Table 4-1, DOCX file.

Whether this mutation affects the activity of the protein is an important question to address. Therefore, we first asked whether the mutation affects the subcellular localization of the protein. When overexpressed as a flag fusion in human embryonic kidney HEK293T cells, we found that the mutated protein remains mainly nuclear (data not shown). Since Dmrta2 functions as a DNA-binding transcription repressor, we then tested the ability of the mutated version to bind DNA by EMSA. To do so, we incubated the radiolabeled Dmrta2 binding site (Dmrta2BS) probe (Murphy et al., 2007), known to bind the Dmrta2, with either nuclear extracts from HEK293T cells transfected with the expression vector of either the DMRTA2^R116P^ or the WT proteins or with either the DMRTA2^R116P^ or the WT proteins produced in vitro in rabbit reticulocyte lysates. We found that the DMRTA2^R116P^ mutated protein is unable to bind DNA (Fig. 4*C*). With this in vitro evidence of the plausible pathogenicity of the *DMRTA2, p.(Arg116Pro) VUS*, this variant can thus be reclassified as likely pathogenic following the updated ACMG criteria (PS3, PM2, PP1, PP3, and PP4). Together, these results suggest that DMRTA2^R116P^ causes cortical malformations by affecting its DNA binding properties.

### Dmrta2 interacts with the NurD complex and Zfp423

**Figure 5.**
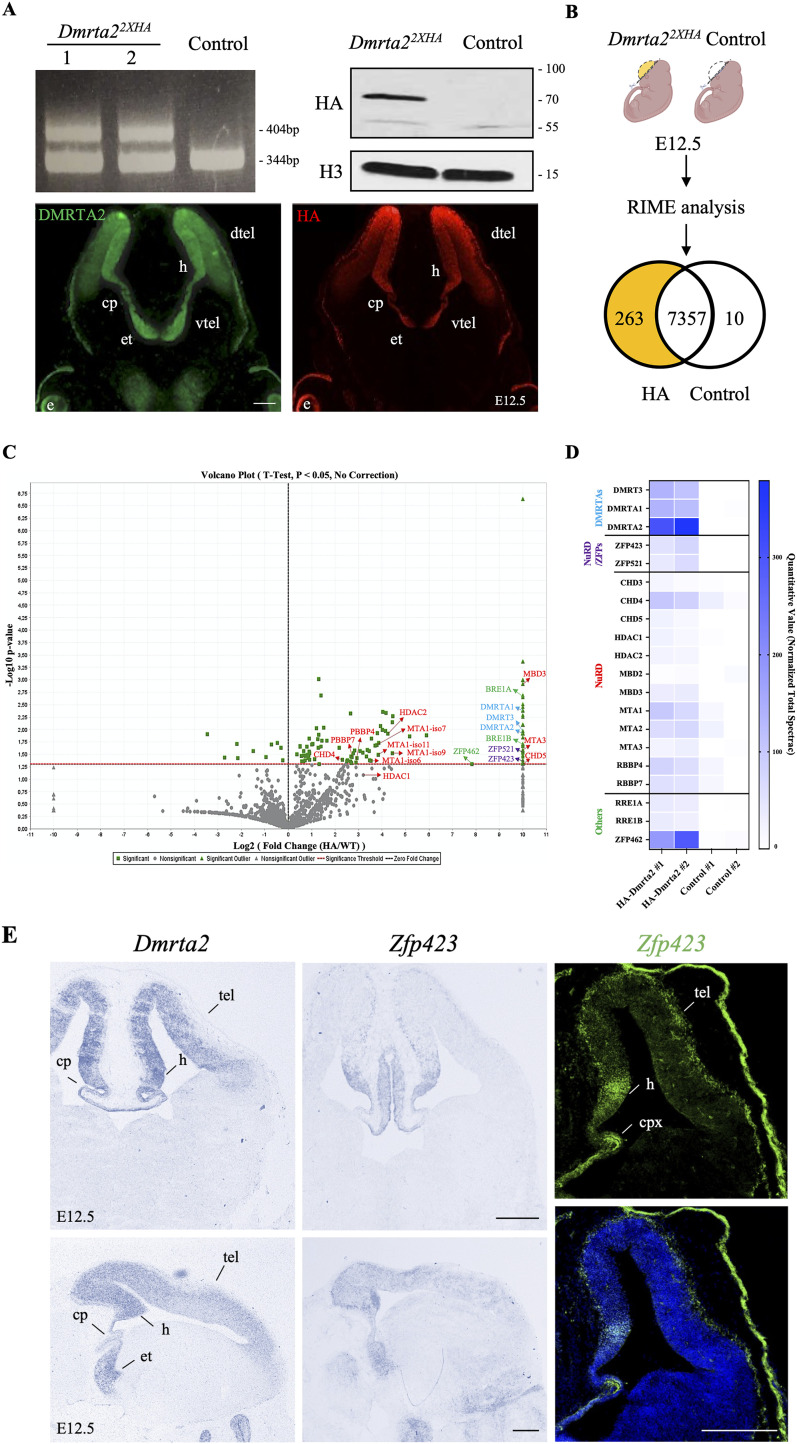
RIME-purified Dmrta2 complexes isolated from dissected cortical tissue contain multiple subunits of NuRD and the NuRD interacting Zfp423 protein. ***A***, Top panels, RT-qPCR using cDNA prepared from dissected cortices of E12.5 embryos with primers flanking the inserted 2× HA sequence, showing that the tagged allele is detectable in the two F0 *Dmrta2^2XHA^* mice (clones 1 and 2) and is absent in WT mice. A Western blot is also shown demonstrating that the HA-tagged protein is expressed in *Dmrta2^2xHA^* mice. Bottom panels, immunostainings showing that the HA-tagged allele is expressed similarly to Dmrta2 in the telencephalon. h, hem; cp, choroid plexus; dtel, dorsal telencephalon; e, eye; et, eminentia thalami; vtel, ventral telencephalon. Scale bar, 200 μm. ***B***, Schematic diagram of the experiment performed. Cortices were dissected from E12.5 *Dmrta2^2xHA^* and control embryos and subjected to RIME analysis. Venn diagrams with the number of the differentially copurified proteins identified in the cortex of *Dmrta2^2xHA^* and control embryos are shown. ***C***, A Volcano plot showing the identified Dmrta2 interacting proteins identified using RIME. All statistically validated proteins are represented by green points, while gray points represent background binding proteins. An arbitrary log2 fold change value of 10 was attributed to the proteins detected only in the HA-Dmrta2 experimental conditions. Proteins of interest are indicated with name (*n* = 3 for each condition). Dmrta2 and other DMRTA family members are highlighted in bright blue, NuRD members in red, and NuRD complex interacting zinc-finger proteins in orange. ***D***, A heatmap showing the significantly enriched proteins identified by RIME and their normalized total spectral count across indicated samples (*n* = 2 for each condition). Peptide coverage for some of the identified Dmrta2 interacting proteins is presented in Extended Data Figure 7-1. ***E***, ISH on coronal (left top panels) and sagittal (left bottom panels) sections of the brain of a E12.5 embryo for *Dmrta2* and *Zfp423* and IF with Zfp423 antibodies on a coronal section through the dorsal telencephalon of a E12.5, without or with DAPI counterstaining (right panels) showing that *Zfp423* is coexpressed with *Dmrta2* in the mouse embryonic cortex. Cp, choroid plexus; et, eminentia thalami; h, hem; tel, telencephalon; Scale bar, 200 μm.

How Dmrta2 represses gene expression is unknown. To identify Dmrta2 interacting partners, we generated transgenic mice expressing Dmrta2 with an N-terminal 2× HA epitope using the CRISPR/Cas9 gene editing system (*Dmrta2^2XHA^*). Figure 5*A* shows RT-qPCR, Western blot, and immunostaining experiments demonstrating that the tagged *Dmrta2* allele is expressed in the telencephalon of *Dmrta2^2XHA^* embryos. We then performed rapid immunoprecipitation followed by mass spectrometry analysis of endogenous protein (RIME) experiments using anti-HA antibodies on chromatin extracts prepared from dissected dorsal cortices from E12.5 *Dmrta2^2XHA^* and WT control embryos. As this method uses formaldehyde fixation to stabilize proteins complexes, it is particularly suited to study chromatin and transcription factor complexes (Mohammed et al., 2016). Among the 263 proteins that selectively copurified with Dmrta2-HA (Fig. 5*B*), the major protein present in the *Dmrta2^2XHA^* sample was Dmrta2 in two independent replicates. Other members of the DmrtA family, such as Dmrt3 and Dmrta1, were also efficiently recovered, which is expected given the ability of DmrtA proteins to bind DNA, forming homo- and heterodimers, trimers, or tetramers (Murphy et al., 2007, 2015). Interestingly, several components of the nucleosome remodeling and deacetylase NuRD complex (including CHD4, HDAC1/2, MBD3, among others; Nitarska et al., 2016) and the vertebrate-specific zinc finger transcription factor Zfp423/ZNF423 (also termed OLF/EBF associated-zinc finger protein OAZ) and its homolog Zfp521 that interact with the NuRD complex (Harder et al., 2014; Li et al., 2021; Shao et al., 2021) and regulate neurogenesis (Cheng et al., 2007; Kamiya et al., 2011; Shen et al., 2011; Ohkubo et al., 2014; Shahbazi et al., 2016; Massimino et al., 2018) were also selectively enriched in the *Dmrta2^2XHA^* samples (Fig. 5*C*,*D*; Extended Data Fig. 5-1). Among the other proteins recovered selectively in the *Dmrta2^2XHA^* sample was the zinc-finger protein Zfp462 which regulates neural lineage specification by targeting the H3K9-specific histone methyltransferase complex G9A/GLP to silence meso-endodermal genes (Yelagandula et al., 2023) and the E3 ubiquitin ligases BRE1a and BRE1b that regulate the cell cycle and differentiation of neural precursor cells (NPCs) through monoubiquitylation of histone H2B (Ishino et al., 2014).

10.1523/ENEURO.0377-24.2025.f5-1 Figure 5-1 **Peptide coverage for some of the Dmrta2 interacting proteins identified with a significance level of P < 0.05 in *Dmrta2^2XHA^* embryos.** Yellow bars represent regions of the full-length protein sequence where peptides were identified, n=2 for each condition. Download Figure 5-1, TIF file.

**Figure 6.**
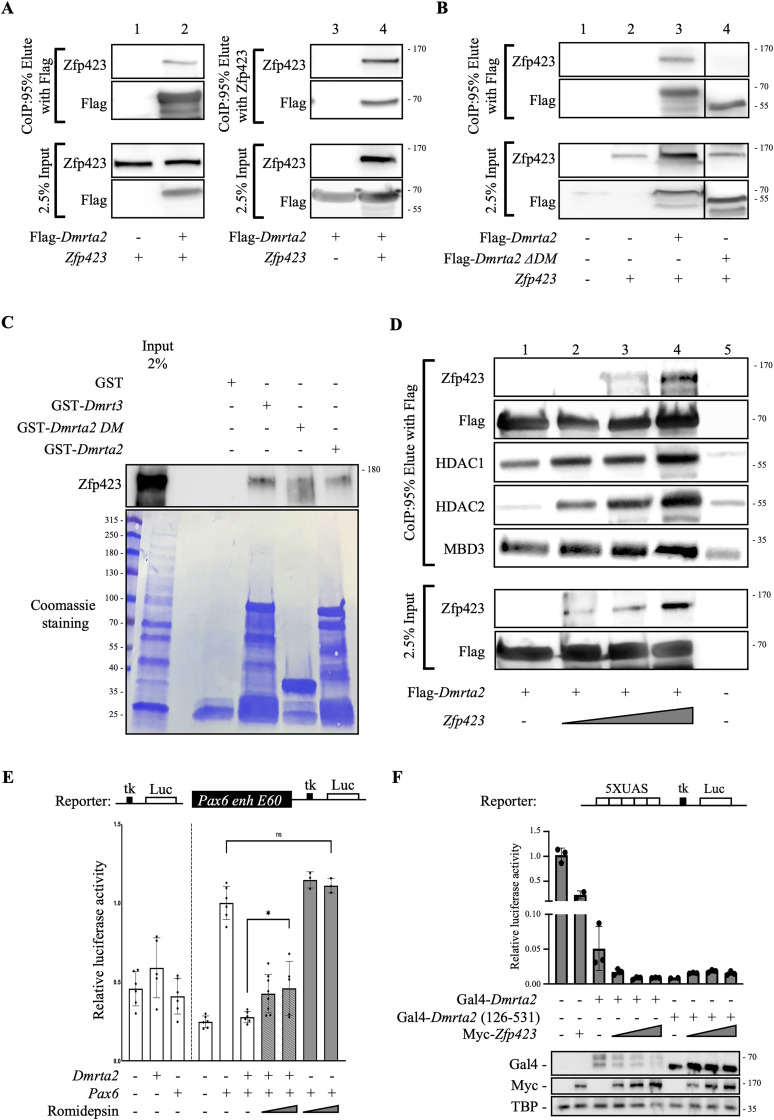
The Dmrta2-Pax6 interaction requires the recruitment of the HDAC-NuRD complex by Zfp423. ***A***, ***B***, Coimmunoprecipitation assays as indicated with Zfp423, Flag-Dmrta2, and a Flag-Dmrta2 mutant lacking the DM domain (Flag-Dmrta2 ΔDM) overexpressed in HEK293T cells as indicated. Note in ***A*** that Flag-Dmrta2 pulled down Zpf423 (line 1–2) and that, conversely, Zpf423 coprecipitated Flag-Dmrta2 (line 3–4). Note in ***B*** that Flag-Dmrta2 ΔDM does not pull down Zfp423 (line 4). *n* = 3. Segments from the same blot have been spliced together to show side by side the results of the WT and the ΔDM Dmrta2 mutant. ***C***, Full-length mouse Myc-tagged Zfp423 was synthesized in vitro and incubated with GST alone or with GST fusion proteins bound to glutathione-agarose beads as indicated. Bound Myc-tagged Zfp423 was detected by immunoblot with an anti-Myc antibody. A 2% input sample was loaded for comparison. The corresponding Coomassie-stained gel is shown. *n* = 2. ***D***, Coimmunoprecipitation assays as indicated with HEK293T cells transfected with a Flag*-Dmrta2* expression construct, alone or together with increasing doses of a *Zfp423* expression construct. Note that Dmrta2 immunoprecipitates some NuRD subunits and that the binding of Zfp423 to Dmrta2 increases the amount of coimmunoprecipitated HDAC1/2 and MBD3. Densitometric quantification of the western blot results is shown in Extended Data Figure 6-1. *n* = 3. ***E***, Reporter assays in P19 cells transfected with a Pax6 E60 tk-luc reporter vector, or an “empty” tk-luc reporter vector as indicated, together with a pCS2Myc*-Pax6* expression vector and/or a pCS2Flag*-Dmrta2* expression vector as indicated, in the absence (white bars) or presence of increasing doses of the HDAC1 inhibitor romidepsin (gray bars). Note that romidepsin leads to a stronger increase of luciferase activity reaching significance in the presence of Dmrta2 but not in its absence. The mean activity of the Pax6 E60 enhancer reporter construct with cotransfected Pax6 is set to 1. NS, not significant. *p < 0.05, one-way ANOVA test. Results of similar reporter assays performed in P19 cells, in the presence or absence of Zfp423 are presented in Extended Data Figure 6-2. ***F***, Reporter assays in HEK293T cells show that both Gal4-Dmrta2 and the Gal4-Dmrta2 (126–531) fusion construct lacking the DM domain required for Zfp423 interaction has strong repression activity on the 5XUAS-tk-luc reporter construct and that Zfp423 slightly increases the repressive activity of Gal4-Dmrta2 but not of the Gal4-Dmrta2 (126–531) construct. In each condition, 200 ng of the 5XUAS-tk-luc reporter was transfected, together with 25 ng of the pCMV-Gal4-Dmrta2 or the pCMV-Gal4-Dmrta2 (126–531) and different doses (200, 400 and 600 ng) of pCDNA3-Myc*-Zfp423* expression plasmids. Values represent the mean ± SD of one transfection done in triplicate. A Western blot showing the expression levels of the overexpressed factors is shown below. Reporter assays showing that the Gal4-Dmrta2 fusion protein represses in a UAS-dependent manner the activity of the 5XUAS-tk-luc reporter are presented in Extended Data Figure 6-3. Reporter assays in HEK293T cells showing that Zfp423 does not increase the modest repression observed when an expression vector encoding the Gal4 DNA-binding domain alone is cotransfected with the 5XUAS-tk-luc reporter are presented in Extended Data Figure 6-4.

Zfp521 can act as a repressor or activator depending on the cellular context (Scicchitano et al., 2019; Chiarella et al., 2021). During the differentiation of embryonic stem cells into neural progenitors, Zfp521 has been shown to act mainly as an activator of neural genes (Kamiya et al., 2011). Zfp462 has been shown to repress mesoendodermal genes during neural differentiation of embryonic stem cells but does not affect *Pax6* upregulation (Yelagandula et al., 2023). Therefore, and based on our data showing that Dmrta2 complexes contain multiple NuRD subunits, we focused on the NurD-interacting Zfp423 protein as a possible Dmrta2 interacting partner potentially involved in its repressive function. By in situ hybridization and immunostaining, we found as previously reported (Cheng et al., 2007; Massimino et al., 2018) that *Zfp423* is expressed in cortical progenitors, the highest expression being detected in the hem region (Fig. 5*E*). To further investigate the ability of Dmrta2 to form a complex with Zfp423, constructs encoding *Zfp423* and Flag-tagged *Dmrta2* were cotransfected in HEK293T cells. In Co-IP assays, results obtained indicated that Flag*-*Dmrta2 interacts with Zfp423 (Fig. 6*A*) and that a Dmrta2 DM domain deletion mutant (Flag-Dmrta2ΔDM) does not bind to Zfp423, revealing that the DM domain of Dmrta2 is required for the interaction (Fig. 6*B*). GST pull-down experiments were also performed using three purified GST-fusion proteins: one encoding the full-length mDmrta2, another the mDmrta2 DM alone, and a third one encoding the related mDmrt3 protein. Mouse Zfp423 has been produced by in vitro transcription/translation and detected by western blot using anti-Zfp423 antibodies. As shown in Figure 6*C*, Zfp423 does not interact with purified GST alone but was recovered with the GST-Dmrta2, GST-Dmrta2 DM, and GST-Dmrt3 fusion proteins. These results confirm the ability of Dmrt transcription factors to interact with Zfp423 and show that the DM domain of Dmrta2 is sufficient for this interaction.

10.1523/ENEURO.0377-24.2025.f6-1 Figure 6-1 **Densitometric quantification of Western blot results of HDAC1 (A), HDAC2 (B), and MBD3 (C) from Figure 9D.** For each comparison, the value for the expression levels in cells transfected with Flag-*Dmrta2* alone is set as 1. The data are presented as a comparison between these baseline cells and those transfected with additional constructs. * P < 0.05, ** P < 0.01, *** P < 0.001, one-way ANOVA test. Download Figure 6-1, TIF file.

10.1523/ENEURO.0377-24.2025.f6-2 Figure 6-2 ***Zfp423* overexpression does not increase the ability of Dmrta2 to repress the activity of the *Pax6 E60*-tk-luc reporter in P19 cells.** Reporter assays in P19 cells transfected with a *Pax6 E60* tk-luc reporter vector, or an ‘empty’ tk-luc reporter vector as indicated, together with a Myc*-Pax6* expression vector with or without 1ng of a Flag*-Dmrta2* expression vector at as indicated, with increasing doses (from 125 ng to 500 ng) of a Myc*-Zfp423* expression vector or a pcDNA3-empty vector as indicated. Values represent the mean+/- SD of one transfection done in triplicate. Ns: not significant, one-way ANOVA test. Download Figure 6-2, TIF file.

10.1523/ENEURO.0377-24.2025.f6-3 Figure 6-3 **A Gal4-Dmrta2 fusion protein represses in a UAS-dependent manner the activity of a 5XUAS-tk-luc reporter.** Results of reporter assays obtained in HEK29T3 cells transfected with an increasing dose of Gal4-Dmrta2 (50, 100, 150, and 200 ng) vector and either an “empty” tk-luc or a reporter possessing five copies of a Gal4 upstream activation sequence (5XUAS) upstream of the tk-luc reporter. Download Figure 6-3, TIF file.

10.1523/ENEURO.0377-24.2025.f6-4 Figure 6-4 **Reporter assays in HEK293T cells showing that Zfp423 does not increase the modest repression observed when an expression vector encoding the Gal4 DNA-binding domain alone is cotransfected with the 5XUAS-tk-luc reporter.** In each condition, 200 ng of the 5XUAS-tk-luc reporter was transfected, together with 25 ng of the pCMV-Gal4-*Dmrta2* or pCMV-Gal4 and different doses of pCDNA3-Myc-*Zfp423* (200, 400, and 600 ng) expression plasmids. Values represent the mean+/- SD of one transfection done in triplicate. Download Figure 6-4, TIF file.

As Zfp423 interacts with the NuRD complex, its interaction with Dmrta2 may contribute to its repressive properties. To test this hypothesis, *Zfp423* was cotransfected at increasing doses with a fixed dose of Flag*-Dmrta2* in HEK293T cells. Co-IP assays were then performed using an anti-Flag antibody followed by Western blotting using antibodies for Flag*-*Dmrta2, Zfp423, and NuRD complex components. Despite some background binding observed with protein G magnetic beads alone used as a negative control, the results obtained show that Dmrta2 immunoprecipitate some NuRD subunits (i.e., HDAC1 and MBD3, compare levels in lines 1 and 5). With increasing doses of Zfp423, as expected, increasing binding of Zfp423 was observed. Interestingly, increased binding of Zfp423 to Dmrta2 appears to increase the amount of coimmunoprecipitated HDAC1/2 and MBD3 (Fig. 6*D*, Extended Data Fig. 6-1). To test the hypothesis that Dmrta2 represses its targets via the recruitment of NuRD components, we performed reporter assays with a *Pax6 E60*-luc reporter construct in P19 cells transfected as above with a *Pax6* expression vector to stimulate *E60* enhancer activity, in the presence or absence of cotransfected *Dmrta2*. Twenty-four hours after transfection, cells were treated with the class I HDAC inhibitor romidepsin, which is known for its selective inhibition of HDAC 1, 2, 3, and 8 (Petrich and Nabhan, 2016; Mayr et al., 2021). Results obtained showed that romidepsin significantly decreases the repressive effect of Dmrta2 and that such an effect is not observed in the absence of Dmrta2 (Fig. 6*E*). These data suggest that Dmrta2 functions as a transcriptional repressor through the recruitment of the NuRD complex.

To determine whether Zfp423 plays a role in Dmrta2 repressive function, we first examine the consequences of its overexpression on the ability of Dmrta2 to repress the E60 enhancer in P19 cells. Cotransfection of *Dmrta2* with an increasing amount of a *Zfp423* expression plasmid did not further decrease luciferase activity (Extended Data Fig. 6-2). As this may be due to the fact that Zfp423 is already strongly expressed in P19 cells (Masserdotti et al., 2010; Cho et al., 2013), to address a potential role for Zfp423 in Dmrta2 repressive function, we turned to HEK293T cells in which *Zfp423* is expressed at low level (proteinatlas.org). As the Pax6 E60 enhancer is not active in HEK293T cells, we performed reporter assays with a luciferase reporter plasmid containing five GAL4 binding sites located upstream of a HSV tk promoter (5XUAS-tk-luc) and an expression construct encoding a chimeric protein consisting of the Gal4 DNA-binding domain fused to Dmrta2, in the presence or absence of MycZfp423. The results obtained (Fig. 6*F*) show that cotransfection of the expression construct encoding Gal4-Dmrta2 decreases luciferase activity, with a low amount of transfected Gal4-Dmrta2 expression vector sufficient to produce a ∼10-fold decrease of luciferase activity. Such a strong decrease was not observed using a luciferase reporter plasmid without GAL4 binding sites upstream of a HSV tk promoter (Extended Data Fig. 6-3). Cotransfection of this Gal4-dependent luciferase reporter with an expression vector encoding Zfp423 at a high dose produced only a weak decrease (∼2-fold). Notably, cotransfection of this Z*fp423* expression vector together with Gal4-Dmrta2 led to a slightly more pronounced decrease than that observed with Gal4-Dmrta2 alone (Fig. 6*F*). Such a cooperative repressive effect on 5XUAS-tk-luc was not observed when Myc-Zfp423 was cotransfected with a Gal4 DNA-binding domain alone expression vector, which we found to slightly decrease luciferase activity (Extended Data Fig. 6-4). As an additional control, to validate the modest cooperative effect observed when Zfp423 is coexpressed with Dmrta2, we also cotransfected Zfp423 with an expression vector encoding a Gal4-Dmrta2 mutant in which the N-terminal part containing the DM domain required for Zfp423 interaction has been deleted, Gal4-Dmrta2 (126–531). Results obtained show that, as observed with Dmrta2 wild type, Gal4DBD-Dmrta2 (126–531) also leads to a strong decrease of 5XUAS-tk luc activity. However, in contrast to the Dmrta2 full-length protein, cotransfection of Zfp423 with this Dmrta2 deletion mutant did not further decrease luciferase activity (Fig. 6*F*). Together, these results confirm the ability of Dmrta2 to mediate transcriptional repression. They reveal that regions downstream of the DM domain of Dmrta2 participate in its repressive properties and suggest that Zfp423 interacting with the DM domain contribute to Dmrta2 repression activity.

## Discussion

In this study, we have investigated how Dmrta2 and Pax6, two transcription factors expressed in the cortical ventricular zone in opposite gradients, interact to control the spatiotemporal identity of neural progenitors and regulate regional neurogenesis. We show that *Pax6* is already upregulated by the loss of *Dmrta2* at E9.5 before the disruption of Wnt signaling, in accordance with its identification as a Dmrta2 direct target (Konno et al., 2019). *Pax6* promotes neurogenesis while *Dmrta2* maintains neural progenitors in the cell cycle (Scardigli et al., 2003; Sansom et al., 2009; Young et al., 2017). We found that the absence of one allele of *Pax6* partially rescues the reduction of the size of the cortex observed in *Dmrta2^−/−^* embryos due to premature neuronal differentiation. This is in accordance with the observation that the siRNA knockdown of *Pax6* expression in medial cortical cells electroporated with siRNAs targeting *Dmrt3* and *Dmrta2* rescues their neurogenic phenotype.

Both Pax6 (Toresson et al., 2000; Yun et al., 2001; Carney et al., 2009; Sun et al., 2015) and Dmrta2 (Desmaris et al., 2018; Konno et al., 2019) have been reported to be required for defining the dorsal telencephalic compartment and directly repress *Gsx1* and *Gsx2*. Pax6 function in dorsoventral telencephalon patterning may also occur through another member of the Dmrt family, Dmrta1, as it is positively regulated by Pax6 and that its overexpression in the ventral telencephalon induces the expression of the dorsal proneural factor *Neurog2* and represses the ventral marker *Ascl1* (Kikkawa et al., 2013). Here we show that the subpallial markers *Gsx2* but also *Gsx1* are robustly ectopically expressed in the abortive cortical primordium of *Pax6; Dmrta2* double KO embryos, a phenotype not observed in single KO, indicating that Dmrta2 and Pax6 cooperation is essential for maintaining cortical identity in dorsal telencephalic progenitors. Such a phenotype is not observed in *Dmrta2^−/−^; Pax6^sey/+^* mutants suggesting that a low amount of Pax6 is sufficient to repress *Gsx1* and *Gsx2* expression. Thus, *Gsx1* and *Gsx2* appear to be particularly sensitive to Pax6, as Gsx2 is to Dmrt factors (Konno et al., 2019). The combined loss of *Pax6* and *Emx2* (Muzio et al., 2002b) and of *Dmrta2* and *Emx2* (Desmaris et al., 2018) also results in the ectopic expression of ventral-specific markers in the dorsal telencephalon. Early telencephalon dorsoventral patterning and the maintenance of the identity of cerebral cortical progenitors appear thus to be under the tight control of multiple cortical factors that cooperate to repress the expression of a set of ventral telencephalic determinants, including Gsx1 and Gsx2.

**Figure 7.**
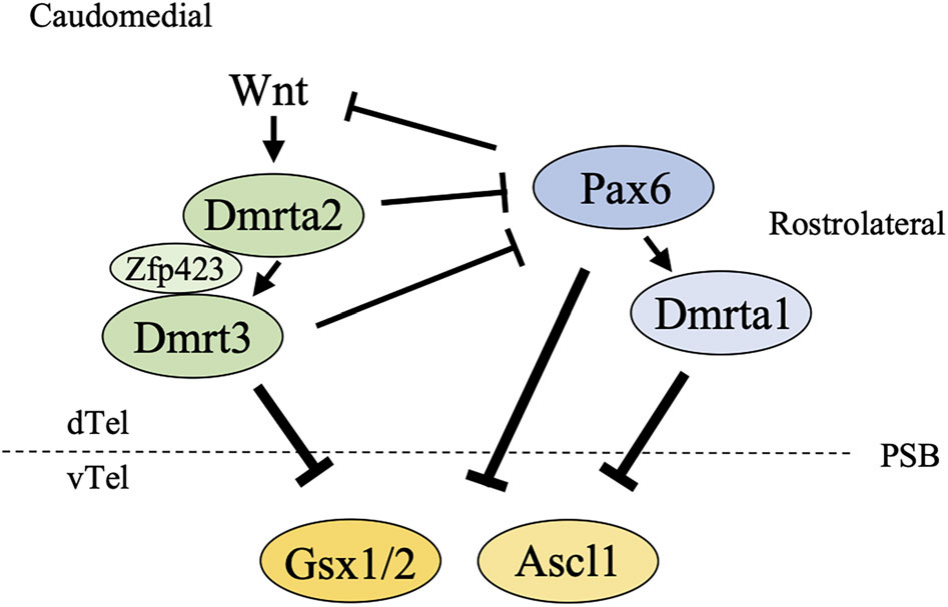
Model illustrating the action of Dmrta2 and related family members in cortical patterning. Our results provide experimental evidence showing that downstream of hem derived Wnt signals, Dmrta2 acts through the repression of *Pax6* to promote caudomedial fate, and it cooperates with Pax6 to repress ventral determinants such as *Gsx1* and *Gsx2*, extending previous studies having defined *Pax6* regulation by Dmrt factors (Konno et al., 2012; Saulnier et al., 2013; Young et al., 2017). In accordance with Konno et al. (2019), the data suggest that Dmrta2 interacting with Zfp423 and the NuRD complex differentially repress the expression of *Pax6* and *Gsx1/2*. Greater or lesser Dmrt repression capacity is indicated by thick or thin repression arrows, respectively. Similarly, *Gsx1* and *Gsx*2 appear more sensitive to Pax6 than *Wnt3a* in the hem. Dmrt3 that is regulated by Dmrta2 contributes to the repression of *Pax6* (De Clercq et al., 2016). *Dmrta1* that is upregulated by Pax6 contributes to its ability to repress ventral telencephalic genes such as *Ascl1* (Kikkawa et al., 2013). PSB, pallium subpallium boundary; dTel, dorsal telencephalon; vTel, ventral telencephalon.

*Pax6* is expressed in a rostrolateral^high^/caudomedial^low^ gradient, opposite to that of *Dmrta2*. *Pax6* regulates dorsoventral patterning in the telencephalon. Within the lateral telencephalon, *Pax6* contributes to the restriction of medial cortical fate (Muzio et al., 2002a; Godbole et al., 2017). *Pax6* also promotes a rostral fate in cortical progenitors. In small eye mutants that lack functional Pax6 protein, caudal visual areas are expanded (Bishop et al., 2000; 2002). The cortex-specific deletion or overexpression of *Pax6* has been also shown to reduce S1 area size (Manuel et al., 2007; Zembrzycki et al., 2013). The study by Konno et al. (2019) suggested that the graded expression of Dmrt factors repressing *Pax6* transcription and thus establishing its lateral^high^/medial^low^ expression gradient provides mediolateral positional information to cortical progenitors. Our findings that the loss of one allele of *Pax6* partially rescues medial cortical hem in *Dmrta2* hetero- and homozygous mutant embryos provide first in vivo experimental evidence supporting this model. It suggests that a higher amount of Pax6 is needed within the cortical primordium to repress hem fate than to repress Gsx2, suggesting that some Pax6 targets are also differentially sensitive to its dosage. In the developing mouse lens, *Six3* expression has been shown to be dose dependent on Pax6 function (Goudreau et al., 2002). Our observation that *Pax6* overexpression rescues the expansion of V1 area observed in *Dmrta2* overexpressing transgenics further demonstrates the importance of the repressive action of Dmrt factors on Pax6 in positional information of cortical progenitors. Together, our data are in agreement with the model proposed by Konno et al. (2019) suggesting Dmrt5 acts mainly by differential suppression of *Pax6* and other homeobox transcription factors. A summary of the interactions identified in this work and in previous studies between Dmrta2 and related family members and Pax6 in cortical patterning is shown in Figure 7. Sexual differences are prevalent in the brain. DMRT transcription factors have been postulated as important determinants of sex differences (Casado-Navarro and Serrano-Saiz, 2022 ). A recent study has shown that one alternative transcript of another member of the DM family, Dmrt2, is slightly more expressed (1.67 times more) in the developing cingulate cortex at E13.5 in males than in females and that its downregulation results in a more pronounced decrease of cingulate cortex neural precursors in male embryos (Bermejo et al., 2025). Although *Dmrta2* has not been identified as a differentially expressed gene showing sex differential expression in recent studies by RNAseq of the developing mouse cortex (Ochi et al., 2024) or during neural differentiation of human embryonic stem cells (Pottmeier et al., 2024), it appears to be transiently expressed at a slightly higher level (1.2×) in males than in females at E11.5. Whether this subtle difference in *Dmrta2* expression between male and female can lead to significant distinct outcomes in cortex development between sexes remains to be explored.

How Dmrta2 represses its targets in cortical progenitors and how it does it in a dose-dependent manner remains today unknown. Therefore, we performed reporter gene assays in P19 cells using the *Pax6 E60* enhancer. Given the low activity of this enhancer in P19 cells, those assays were done in the presence of *Pax6*, as previous studies reported that Pax6 autoregulates its expression (Aota et al., 2003; Manuel et al., 2007, 2015) and that Pax6 binds to this enhancer region (Sun et al., 2015 ). In accordance with these studies, our results show that Pax6 increases luciferase activity using this *E60* enhancer-driven reporter construct. However, this is not in agreement with the observation that this *E60* enhancer remains active in *Pax6*^sey/sey^ mutants (McBride et al., 2011). The reason for this discrepancy remains unclear. Our results indicate that Dmrta2 counteracts the activation by Pax6 of this *E60* enhancer-driven reporter and that the zinc finger motifs of Dmrta2 that are required for DNA binding (Murphy et al., 2015) are essential for this repression. These results suggest that Dmrta2 acts as a DNA-binding repressor on the *Pax6* locus, as it appears to be the case on the repressor of neurogenesis *Hes1* and on the *Gsx2* genomic loci (Young et al., 2017; Desmaris et al., 2018). This is also further supported by the identification of the human DMRTA2^R116P^point mutation reported here that, to our knowledge, is the only mutation in DMRTA2 suggested to play a role in microcephaly by affecting its ability to bind DNA. The DMRTA2^R116P^ mutation corresponds to DMRT1^R/K122^ involved in DNA phosphate backbone contacts. The fact that the change is to a proline, a helix-breaking residue, and occurs close to DMRT1^R123^ that makes base-specific contacts likely explains the observed disruption of DNA binding. An animal model of the DMRTA2^R116^ mutation may help to elucidate the cause of the phenotype observed in this family.

One critical step in the understanding of the mode of action of a transcription factor is the identification of the protein complexes in which it acts. Our data indicate that Dmrta2 interacts with the NuRD complex and with the multizinc finger Zfp423 and related Zfp521 proteins that are expressed in cortical progenitors (Kamiya et al., 2011; Massimino et al., 2018) and themselves interact with NuRD components (Harder et al., 2014; Li et al., 2021; Shao et al., 2021). They show that Dmrta2 associates with Zfp423 via its DM domain and suggest that the recruitment of Zfp423 modestly contributes to Dmrta2's repressive properties. We found however that regions downstream of the DM domain of Dmrta2 also acts as a repressor domain, indicating that Zfp423 although contributing to is not obligatory for Dmrta2 repressive properties.

Although further studies are needed to demonstrate that Zfp423 and related Zfp521 interact with Dmrta2 in cortical progenitors and help it to repress some of its targets, they appear as potential candidate Dmrt interacting partners. Indeed, Zfp423 and Zfp521 both play important roles in neural development. The importance of Zfp521 for neural development is demonstrated by the fact that it is essential and sufficient for driving the intrinsic neural differentiation of mouse ES cells (Kamiya et al., 2011; Shahbazi et al., 2016). ZNF423 mutations are associated with Joubert Syndrome, a ciliopathy causing cerebellar vermis hypoplasia and ataxia. Null Zfp423 mutants develop cerebellar malformations and hindbrain choroid plexus hypoplasia (Cheng et al., 2007; Casoni et al., 2017, 2020). In the forebrain, the corpus callosum is absent and the hippocampus is reduced. The cortex is thinner than the controls due to reduced proliferation. When Zfp423 is overexpressed in the cortex of E13.5 mouse embryos, it increases the number of electroporated cells positive for the neuronal marker *Tubb3* at the expense of mitotically active PAX6^+^ radial glia cells (Alcaraz et al., 2006; Massimino et al., 2018). Whether these changes in cell proliferation and differentiation upon manipulation of Zpf423 expression are linked to its interaction with Dmrta2 or, alternatively, to its ability to modulate other signaling pathways, like the SMAD/BMP, NOTCH, and SHH pathways (Hata et al., 2000; Ku et al., 2006; Masserdotti et al., 2010; Hong and Hamilton, 2016), remains to be elucidated.

## Data Availability

All data used in the preparation of this manuscript will be provided upon request.

## Synthesis

Reviewing Editor: Benjamin Deneen, Baylor College of Medicine Decisions are customarily a result of the Reviewing Editor and the peer reviewers coming together and discussing their recommendations until a consensus is reached. When revisions are invited, a fact-based synthesis statement explaining their decision and outlining what is needed to prepare a revision will be listed below. The following reviewer(s) agreed to reveal their identity: Noriko Osumi.

While the reviewers are generally positive about the manuscript, there are several revisions that need to be implemented in order for us to proceed with this article. Rev 1 is asking for additional clarifications on cell lines, sex of animals, and justification of the source of mutations in the human populations. Rev 2 has more substantive requests that also pertain to clarifications and some issues relating to statistics and quantification.

## Author Response

Answer to Reviewers' comments:

Here is a description of the changes made in response to the reviewer's comments.

Reviewer 1 Major comments 1) According to the current knowledge, gene expression profiles are different between male and female even in the embryonic stage (Ochi et al., Sci Data, 2024). Therefore, the sex of the embryos used in the experiments should ideally be analyzed and indicated for all samples.

We thank the reviewer for this relevant comment. Indeed, sexual differences are prevalent in the brain and DMRT transcription factors have been postulated as important determinants of sex differences (Casado-Navarro and Serrano-Saiz, 2022). However, when we have started this study a few years ago, we didn't systematically consider the two sexes separately, as no study had reported gender differences in gene expression in the cerebral cortex. At this stage, this is unfortunately no longer possible. The fact that non-sex-determined embryos were used in all our analyses is now mentioned on page 6 in the revised version of our manuscript. In the discussion section, we now mentioned (page 32) this issue in the light of recent work on the subject.

2) P19 cells are derived from male teratocarcinoma, and HEK293T cells are from female embryonic kidney samples. What are the scientific reasons that the author used P19 cells for dual-luciferase reporter assays and HEK293T for EMSA? We have used P19 cells for our reporter assays as they have been used extensively as model for in vitro studies on neurogenesis, including for studies on transcription factors such as Pax6 and Emx2 in cortical progenitors, as they express Sox2 but are negative for Pax6 and Emx2 which allows to test the effect of adding them on the function of the enhancers under study (see for example Mariani et al., 2012; Pattabiraman et al., 2014). This is now mentioned on page 23 of the revised manuscript.

Co-IP, EMSA and UAS-dependent luciferase reporter assays that were performed during the revisions were done in HEK293T cells. HEK293T cells were chosen here not as a model for neurogenesis but because they can be transfected efficiently and thus achieve a high level of expression of the transfected genes.

3) In mammalian corticogenesis, Pax6 regulates another family of Dmrt genes, Dmrta1 (Kikkawa et al., Genes Cells, 2013); expression of Dmrta2 is not changed in the Pax6-/- rat telencephalon. Is there a mutual interaction of Dmrta1 and Dmrta2? Our previous results have indicated that in the developing cortex of E12.5 Dmrt3 single KO, Dmrta2 single KO and Dmrt3/Dmrta2 double KO embryos, Dmrta1 like Pax6 is upregulated (Desmaris et al., 2018). This is in accordance with Dmrta1 being an upregulated downstream target of Pax6. This is now mentioned in the discussion of our revised manuscript (page 30).

4) How is it possible to attribute the mutation of DMRTA1 gene to a Pakistani case of microcephaly with a simplified gyral pattern along with agenesis of the corpus callosum and colpocephaly? Dmrta2-/- mice also lack the corpus callosum and colpocephaly? Microcephaly with agenesis of the corpus callosum is exactly the phenotype observed in Dmrta2-/- mice (Saulnier et al., 2012). Colpocephaly, the selective dilatation of the occipital horns of the lateral ventricles is a symptom usually diagnosed early after birth, associated with agenesis or dysgenesis of the corpus callosum. Although not observed in Dmrt5-/- embryos (which die at birth), this abnormality of the occipital part of the brain is also consistent with Dmrt5 caudomedialhigh/ rostrolaterallow gradient of expression. The similarity/relationship of the phenotype observed in human with that observed in the mouse is now mentioned in the result section of the revised manuscript (pages 24,25).

5) It would be better to provide a graphical summary for the interaction of Dmrta2 and other related molecules.

A graphical summary of the expression and interaction of Dmrta2 and other family members with Pax6 in cortical patterning is now provided in Figure 10. This figure is referenced in the discussion (page 32) of the revised manuscript.

Minor issues:

1) What is the strain of genome-edited Dmrta2 knock-in mice? It is written that B6D2F2 zygotes were used for injection, but in which strain the following mice are maintained and how many generations of the genome-edited mice were used for this study? The authors should also describe how the embryonic day is determined in Methods.

Founder transgenic mice were crossed with C57BL/6J and selected founders were then maintained in this background for at least three generations before being used in this study. This is now indicated on page 6 in the MM of the revised manuscript.

How embryonic days are determined was already mentioned in the initial submitted manuscript and probably went unnoticed (please see the MM, section, "animal lines and genotyping", page 5, lines 1-2).

2) The subtitle "Immunofluorescence and in situ Hybridization" in Methods should read "Immunofluorescence and in situ hybridization". Grammatical check should be performed by a native editor throughout the manuscript.

The correction in this subtitle has been done. The entire manuscript has been proofreaded.

3) It would be reasonable to separate the section of cell culture and other assays because in the dual reporter assay, only P19 cells were used. Also, abbreviation of HEK239T should be indicated.

The section of cell culture has been separated from the other assays. The abbreviation of HEK239T is now indicated (see page 11 in the MM of the revised manuscript).

4) There is no statement of how to measure the cortical size shown in Figure 1. In addition, the title of the figure legend "Reduction of Pax6 partially rescues telencephalic vesicle growth in Dmrta2-/- embryos" is somehow misleading.

How the quantification of the cortical hemisphere and area size has been performed is now described (see page 8 in the MM of the revised manuscript).

In the revised manuscript, page 46, the title of Fig.1 has been changed to: "Reduction of the size of the cerebral hemisphere observed in Dmrta2-/- embryos is less severe in the absence of one allele of Pax6". A sentence has been added in the result section of the revised manuscript to conclude about this observation (page 21, lines 2-3).

5) All the images of embryonic/postnatal brains and sections lack scale bars.

Scale bars have been added in all images of embryonic/postnatal brains and sections.

Reviewer 2 1)Overall, the experiments that define Pax6 regulation by Dmrta2 are incremental. Young 2017 and Konno 2019 (among others) postulate that Dmrta2 regulates pax6 expression. Konoo 2019 also shows that Dmrta2 binds to the pax6 promoter. While the luciferase result is this manuscript is new, it is not a significant advancement.There is a general lack of thorough examination of the phenotypes in Fig 1-4 and associated supplemental figures. For example, in Figure 1 whole brains are shown but cross sections are not presented. Nor are markers used to define the phenotype (like organization of the cortex). Numerous markers such as Tbr1, Brn2, Sabt2, Ctip2, Gsx2, Gad2, etc should have been analyzed to provide consistent comparison to previous publications.

We agree that the idea that Dmrta2 regulates cortical development via Pax6 is not new, but we felt that this idea still needed to be demonstrated. In particular, the fact that Dmrta2 regulates cortical regionalization via Pax6 had not been demonstrated. It is with this in mind that we analyzed the phenotype of Dmrt5-/- embryos in the absence of one or two Pax6 alleles. The aim was thus not to complete a full histological and molecular analysis of all single and hybrid phenotypes, which would have been a considerable task, also in view of the difficulty of obtaining double KO embryos.

2)Similarly Fig 2 should have been further analyzed for other markers that define the boundaries. For Figure 2, Dmrta2-/- Pax6sey/+ mutants showed very similar Gsx2 expression to the Dmrta2-/- single mutant.

We have now analysed Gsx1 as the ChIP-seq data of Konno et al., 2019 identified a conserved non coding region bound by Dmrta2 in the Gsx1 locus. Gsx1 is expressed in MGE and vLGE in WT embryos. We found that like Gsx2, it only robustly expands into the cortical primordium in the double KO embryos. This result that further show that Dmrta2 and Pax6 are both required and cooperate in maintaining cortical identity in dorsal telencephalic progenitors is now mentioned on pages 20-21 in the revised manuscript. It has been included in an additional supplemental figure (Extended data fig. 2.1). We also found that Dmrta2 represses the enhancer activity of a tk-reporter construct under the control of this Gsx1 regulatory element in P19 cells. This observation is now mentioned on page 24 in the revised manuscript. The results have been included in an additional supplemental figure (Extended data Fig. 5-3).

The Gsx2 phenotype in Fig2 is now better described. The fact that in contrast to double KO embryos, in Dmrta2-/-;Pax6sey/+ mutants, Gsx2 expression only very slightly crossed the pallium-subpallium boundary, as in the single mutants, is now mentioned (page 20 in the revised manuscript).

3) In Fig1 and 3A, absence of one Pax6 allele partially rescues the Dmrta2 knockout, but for Gsx2, this does not seem to be the case. Is there a way to quantify the phenotype and comment on this discrepancy? We thank the reviewer for prompting us to better describe the phenotype observed in Dmrta2 mutants upon the loss of one or both Pax6 alleles. Indeed, the phenotype that we observed for ventral determinants such as Gsx2 is distinct from that of genes expressed in the cortical hem or that observed on the size of the hemispheres. The fact that Gsx2 and Gsx1 robustly expands into the cortical primordium only in the double KO embryos is now clarified on pages 20-21 of the revised manuscript. We believe this discrepancy is due to the fact that Pax6 targets such as Gsx2 and Gsx1 are particularly sensitive to Pax6. This is now discussed (page 30) of the revised version of our manuscript.

4) For Fig 3B, although the phenotype is drastically different between certain mutant conditions, it would be helpful to use another marker to highlight expression of Wnt8b in different regions, to substantiate the claim that "Wnt8b expression appeared to extend slightly more dorsally in the reduced medial telencephalon than in Dmrta2-/- embryos; and appeared, in contrast, more restricted in the abortive cortex of Dmrta2-/-; Pax6sey/sey embryos." We agree it would have been useful for the Wnt8b marker to distinguish its expression in the medial telencephalon versus the eminentia thalami. However due to the limited number of double KO we have been able to collect, we weren't able to do it. As the Dmrta2 and Pax6 mouse mutant lines are no longer available in the lab (research on cortex development coming to an end in my lab), we have been unfortunately unable to carry out this experiment during the revisions. However, we have now quantified the extent of Wnt8b expression domain by measuring its length in the medial telencephalic neuroepithelium of Dmrta2+/- versus Dmrta2+/-;Pax6Sey/+. In the images shown, the extent of the hem region itself (as apparent by the more intense Wnt8b expression) that is longer in Dmrta2+/-; Pax6sey/+ and in Dmrta2+/-; Pax6sey/sey embryos than in Dmrta2+/- is now indicated. To improve clarity, the entire paragraph describing Wnt3a and Wnt8b expression observed in each genotype (pages 21-22 of the revised article) has been rewritten. The data on Wnt8b are now shown separately in a supplemental figure (Extended data Fig. 3-1). In this figure, we have only retained data on heterozygous Dmrta2 mutants in which Wnt8b remains detectable in the telencephalon and discernable in the hem.

4) Statistics should have included Anova analysis.

Anova analysis has been performed for data shown in Figure 5, 9 and extended fig.5-3, 9-1 and 9-2.

5) Please provide a rationale for why a teratocarcinoma cell line was used for the experiments, as opposed to HEK cells, which the authors used to overexpress the Dmrta2 as a flag fusion.

Please see our answer to comment #2 of reviewer 1.

6) In the discussion, the authors write: "In this study, we provided an extensive analysis of how Dmrta2 and Pax6 interact to control the spatiotemporal identity of neural progenitors and regulate regional neurogenesis." This statement is not backed up by data presented in the manuscript. To state this, analysis of spatiotemporal identity of neural progenitors with different conditional mutants needs to be examined, or this statement needs to be adjusted to reflect the presented data.

As suggested, the sentence has been modified (see page 30 of the revised manuscript).

7) The statement in the abstract "Dmrta2 binds to the Zfp423 zinc finger protein and that it enhances its ability to recruit the NurD repressor complex" is also not substantiated by the data.

To confirm the ability of Dmrt5 to interact with Zfp423, we performed GST pull-down experiments. Results obtained confirm this interaction. They are described on page 27 and shown in Fig. 9C of the revised manuscript.

We agree with the referee. As requested, this statement has been removed everywhere in our revised manuscript. In the discussion (page 33), based on the results obtained during the revision using UAS dependent luc reporter and Gal4-Dmrta2 fusions (see answer to comment #9), we now emphasize that, although Zfp423 seems capable of contributing to the repressive properties of Dmrta2, it is by no means required.

8) The interaction between Dmrta2 and Zfp423 was confirmed in cultured cells, but the ability of this interaction to recruit and repressor complex was not shown. It is odd that Figure 7d does not show HDAC1/2 as Mass Spec interactors of Dmrta2 but the authors used these markers for CO-IP validation of NuRD complex in figure8. Is there a reason why NuRD components identified by Mass Spec were not tested? Also, endogenous Co-IP from developing cortex should be included to provide convincing results in addition to the Co-IP from cell lines.

In fact, HDAC1/2 and other component of the NuRD complex have been identified as Mass Spec interactors of Dmrta2. This is visible in Fig 7C,D and in Extended data Fig.7-1 of the revised manuscript. This is the reason why we tested them in the CO-IP in Fig. 8.

As the Dmrta22XH transgenic line is not available anymore in the lab, we unfortunately have not been able to perform the requested endogenous Co-IP from the developing cortex during the revision. We however believe that our RIME data performed on dissected dorsal cortices from E12.5 Dmrta22XHA and WT control embryos strongly suggest that the interaction occurs in cortical cells.

9) Fig 8d shows a change in enhancer activity with increasing concentrations of romidepsin. This analysis will need to include co- transfection of Dmrta2 and Zfp423 to validate that together, they repress enhancer activity. Additionally, the effect of romidepsin should be tested in the absence of Dmrta2-flag to confirm that there are no off-target effects leading to the increase in luciferase activity.

We thank the referee for prompting us to further analyze the ability of Dmrta2 and Zfp423 to repress together enhancer activity. As requested, we have tested whether coexpression of Zfp423 together with Dmrta2 helps it to repress the E60 enhancer in P19 cells. Our results (Extended data Fig.9-2) show that increasing Zfp423 does not lead to a stronger decrease of luciferase activity, perhaps due to the fact that Zfp423 is already highly expressed in P19 cells (Cho et al., Plos One 2013). Therefore, to address a potential role for Zfp423 in Dmrta2 repressive function, we performed reporter assays in HEK293T cells in which Zfp423 is expressed at low level. As the Pax6 E60 enhancer is not active in HEK293T cells, we transfected these cells with an expression construct encoding a chimeric protein consisting of the Gal4 DNA-binding domain fused to Dmrta2 and a luciferase reporter plasmid containing five GAL4 binding sites located upstream of a HSV tk promoter in the presence or absence of Zfp423. The results obtained confirm the ability of Dmrta2 to mediate transcriptional repression and suggest that Zfp423 can contribute, modestly, to Dmrta2 repression activity. They also reveal that regions downstream of the DM domain of Dmrta2 also contribute to its repressive function. These results shown in Fig. 9F and Extended data Fig.9-3 and 9-4 are described page 28-29 in the result section and page 33 in the discussion of the revised manuscript.

As requested, we have also repeated the experiment with romidepsin, now in the presence but also in the absence of Dmrta2. The results show that romidepsin significantly decreases the repressive effect of Dmrta2 and that such an effect is not observed in its absence. These data are shown in Fig9E and described page 28 of the revised manuscript.

Minor points 10) In figure 7 co-localization images for A and F would be helpful for interpretation. The zfp243 immunostaining is difficult to see in the current image.

These images have been enlarged and the contrast improved.

11) There are several grammatical errors that distract from the reading of the manuscript.

The entire article has now been proofreaded.

12) There is a general lack of labeling in figures and a lack of spatial guides on figures to orient the reader to the location that is being analyzed.

Additional annotations have been added in some figures (i.e Fig. 3) to help the reader.
