# Supplementary figures and images for: Evidence That Dmrta2 Acts through Repression of Pax6 in Cortical Patterning and Identification of a Mutation Impairing DNA Recognition Associated with Microcephaly in Human
Source: eNeuro. 2025 Jun 13;12(6):ENEURO.0377-24.2025. doi: 10.1523/ENEURO.0377-24.2025 (PMC12186615; doi:10.1523/ENEURO.0377-24.2025)

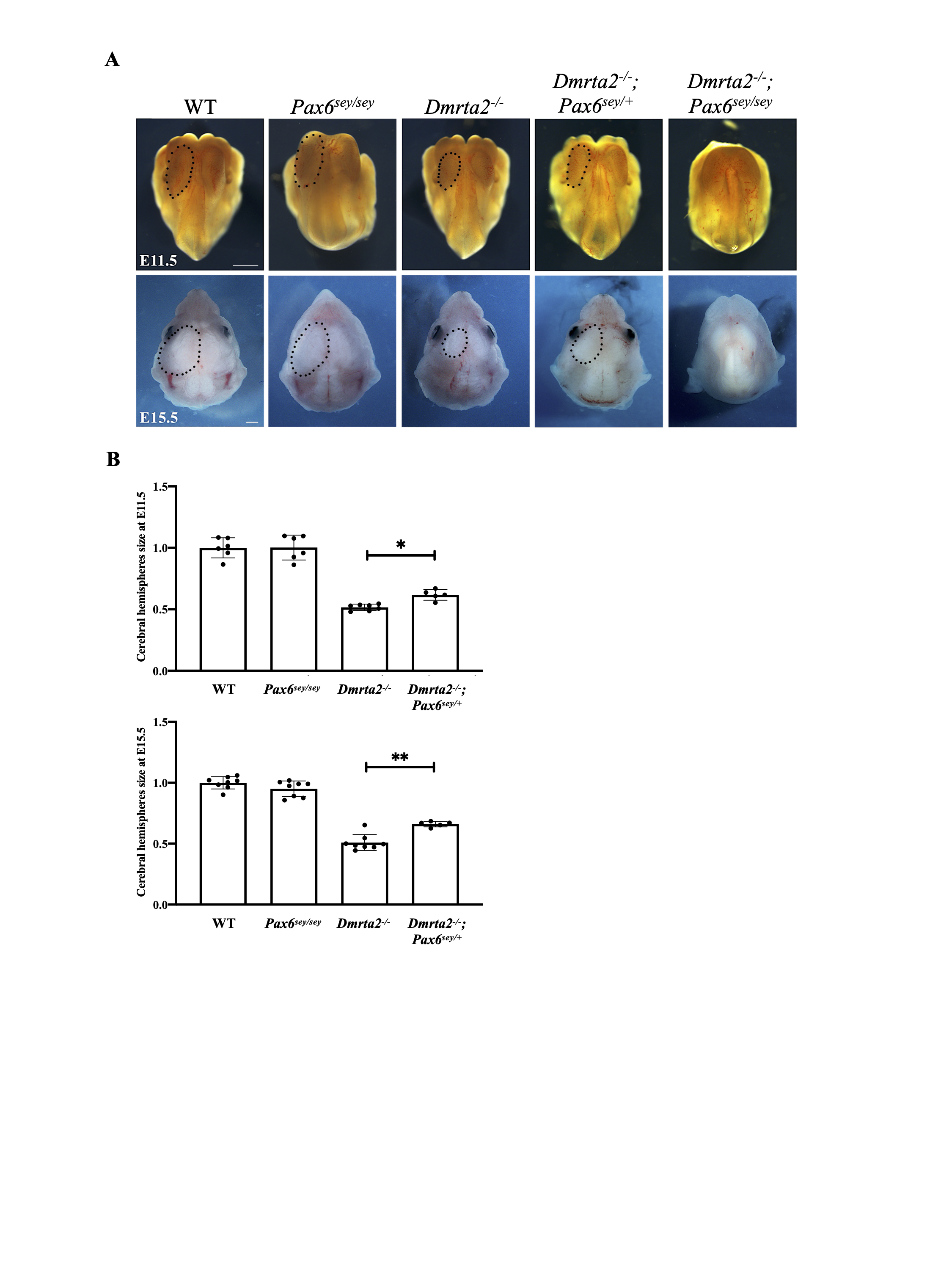

Supplement: Figure 1-1 — The growth of the telencephalic vesicles is rescued upon the loss of one Pax6 allele in Dmrta2-/- embryos. (A) Dorsal views of the brain of E11.5 and E15.5 embryos of the indicated genotype, Scale bar, 100μm. (B) Graphs representing the surface area of E11.5 and E15.5 cerebral hemispheres compared to WT set to 1.*P < 0.05, **P < 0.01, Student’s t-test. Download Figure 1-1, TIF file. [file eneuro-12-ENEURO.0377-24.2025-s003.tif]

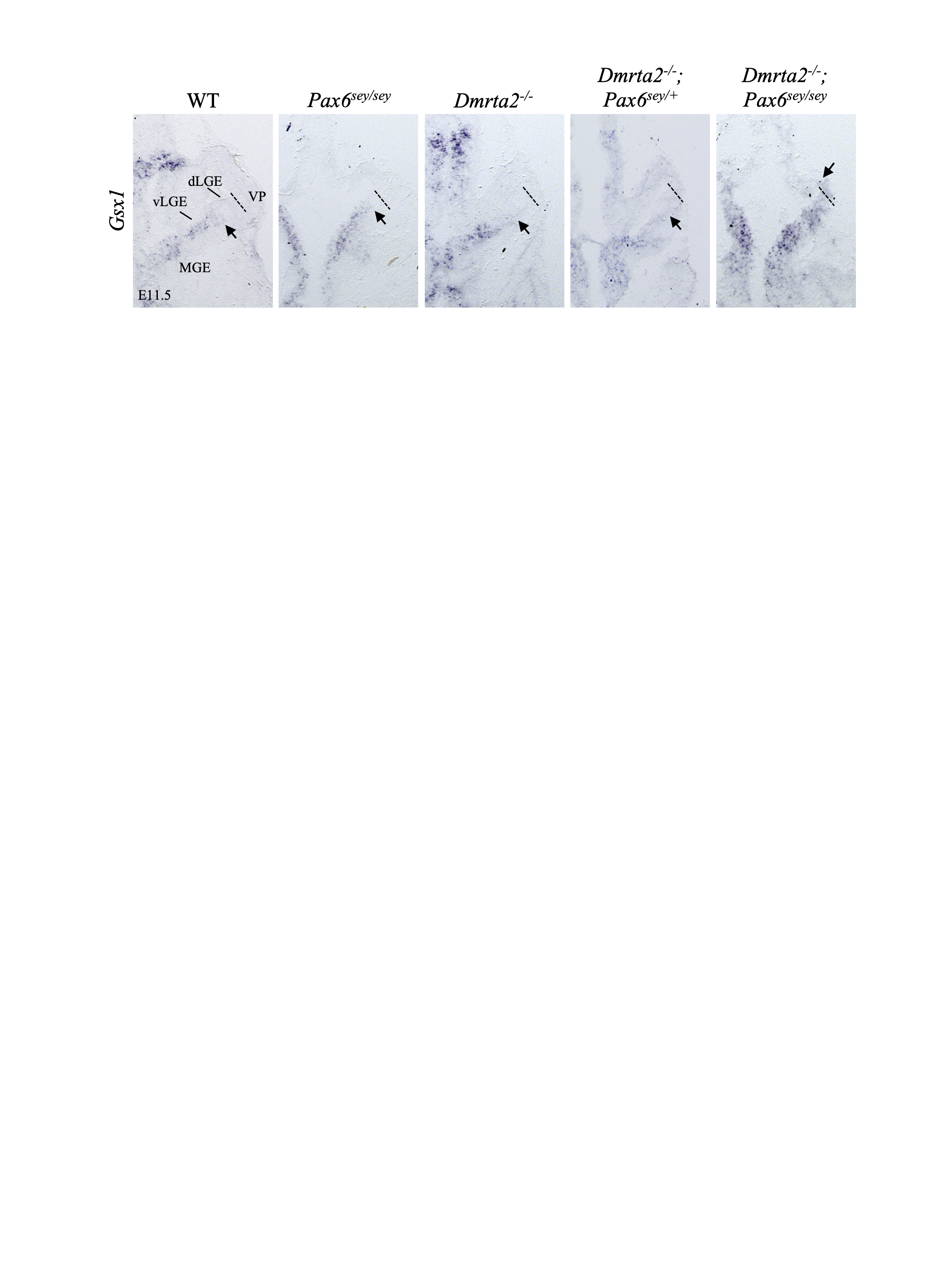

Supplement: Figure 1-2 — The ventral determinant Gsx1 expands dorsally in the abortive cortical primordium of Dmrta2-/-; Pax6Sey/Sey embryos. Coronal brain sections through the brain of E11.5 embryos of the indicated genotypes processed by ISH for Gsx1. Note that Gsx1 expression remains restricted to the ventral telencephalon in Pax6sey/sey, Dmrta2-/- and Dmrta2-/-; Pax6sey/+ embryos but appears to expand dorsally into the telencephalon in Dmrta2-/-; Pax6sey/sey embryos. Arrows point to the dorsal limit of Gsx1 expression. The pallium-subpallium (PSB) boundary region is indicated by a dashed line. Abbreviations: MGE, medial ganglionic eminence; PSB, pallium/subpallium boundary; vLGE, ventral lateral ganglionic eminence; VP, ventral pallium. Download Figure 1-2, TIF file. [file eneuro-12-ENEURO.0377-24.2025-s004.tif]

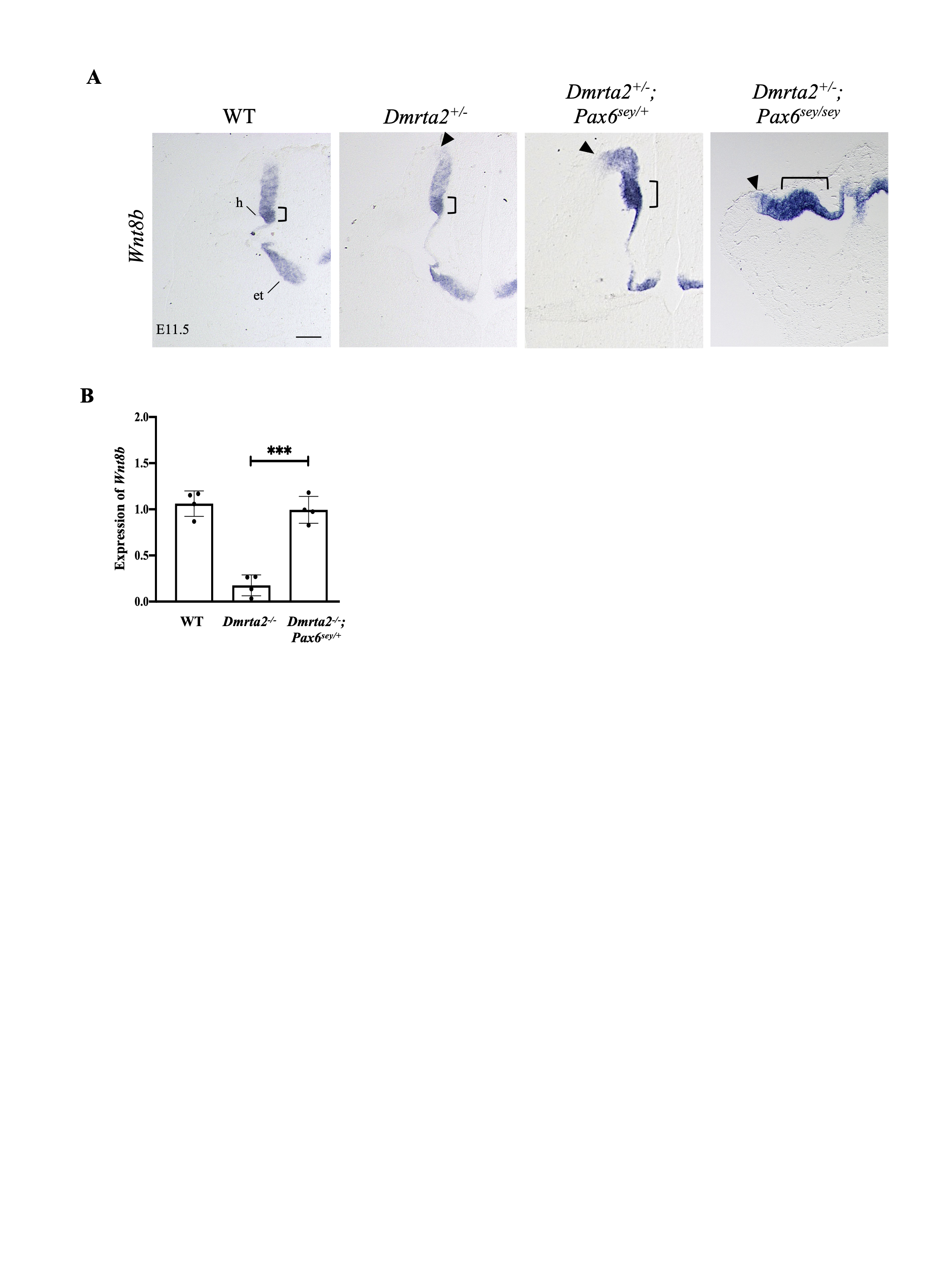

Supplement: Figure 1-3 — Reduction of Pax6 partially rescues medial cortical fate in Dmrta2 homo- and heterozygous mutant embryos. (A) Coronal sections through the brain of E11.5 embryos of the indicated genotype processed by ISH for the expression of Wnt8b marking the dorsomedial telencephalic primordium. Arrowheads indicate the dorsal extent of Wnt8b expression detected in the pallium. The extent of the hem as revealed by more intense Wnt8b expression in Dmrta2+/- and in Dmrta2+/-; Pax6sey/+ is indicated by brackets. Et: eminentia thalami; h, hem. Scale bar, 500 μm. (B) Quantitative RT–qPCR analysis of Wnt8b in dissected cortices of Dmrta2-/-, Dmrta2-/-; Pax6sey/+ and WT control embryos is shown on the left. Results are normalized to the level of the expression detected in the cortex of WT embryos. ***p < 0.001, Student’s t-test. Download Figure 1-3, TIF file. [file eneuro-12-ENEURO.0377-24.2025-s005.tif]

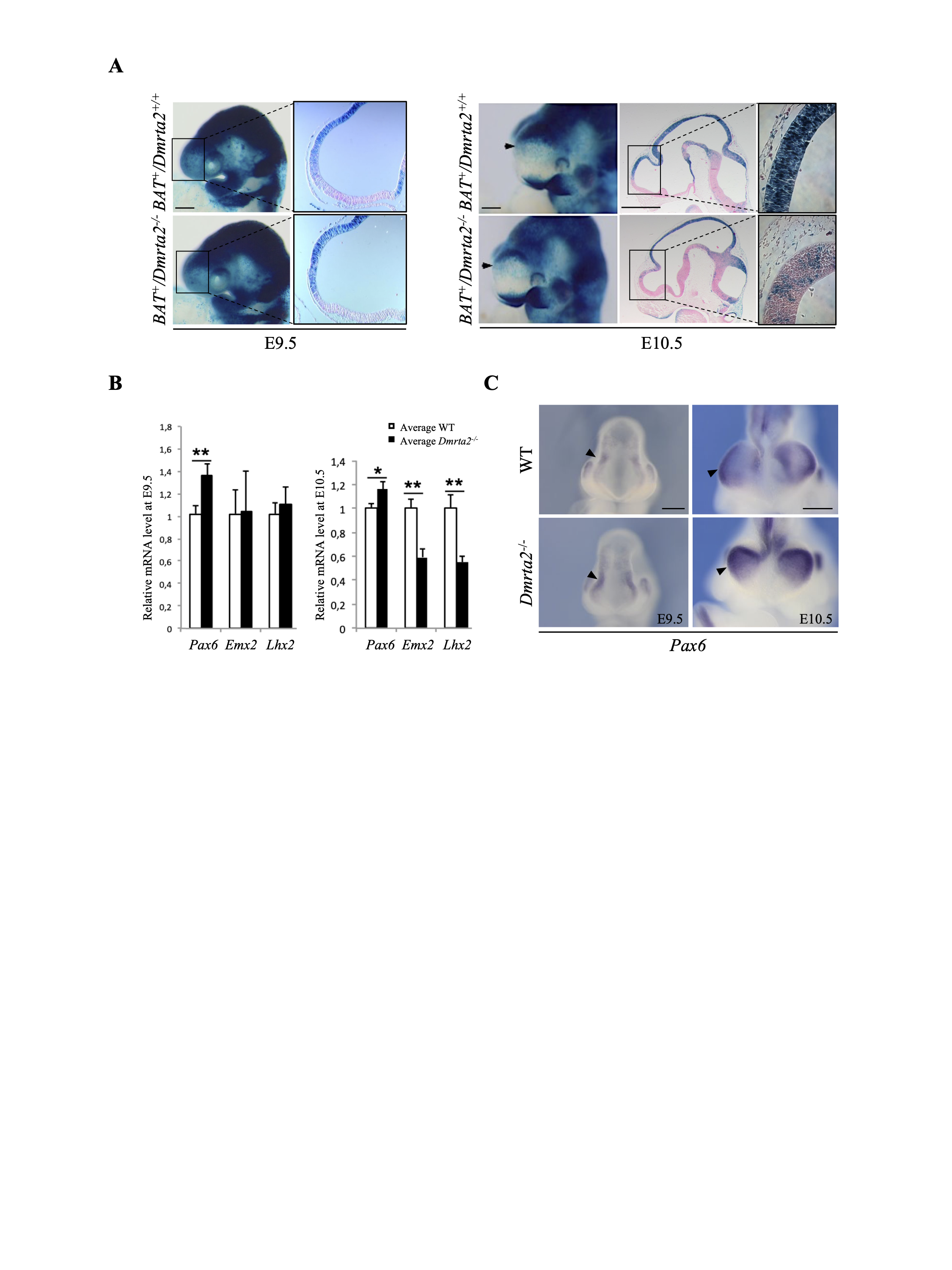

Supplement: Figure 3-1 — Pax6 is already upregulated by the loss of Dmrta2 at E9.5 before the Wnt signaling pathway appears to be affected. (A) Whole-mount X-gal staining on BAT+/Dmrta2+/+ and BAT+/Dmrta2-/- embryos at E9.5 and E10.5. Note on the whole-mount views (arrowheads) and on the sections and magnification views shown on the left that the staining detected in BAT+/Dmrta2-/- embryos is similar to that of BAT+/Dmrta2 +/+ embryos at E9.5 but is reduced at E10.5. Scale bar, 200 μm. (B) RT–qPCR analysis of Pax6, Emx2, and Lhx2 expression in the cortex of Dmrta2-/- and WT embryos. Results are normalized to the level of expression in the cortex of WT embryos. Note that Pax6 is already upregulated in the cortex of Dmrta2-/- at E9.5, which is not the case for Emx2 and Lhx2, whose deregulation is only observed from E10.5. Error bars indicate SDs of at least three independent experiments. *P <0.05 ,**P < 0.01. (C) Whole mount ISH analysis of Pax6 expression shows that its upregulation (arrowheads) in Dmrta2-/- embryos can already be seen from E9.5. Scale bar, 200 μm. Download Figure 3-1, TIF file. [file eneuro-12-ENEURO.0377-24.2025-s006.tif]

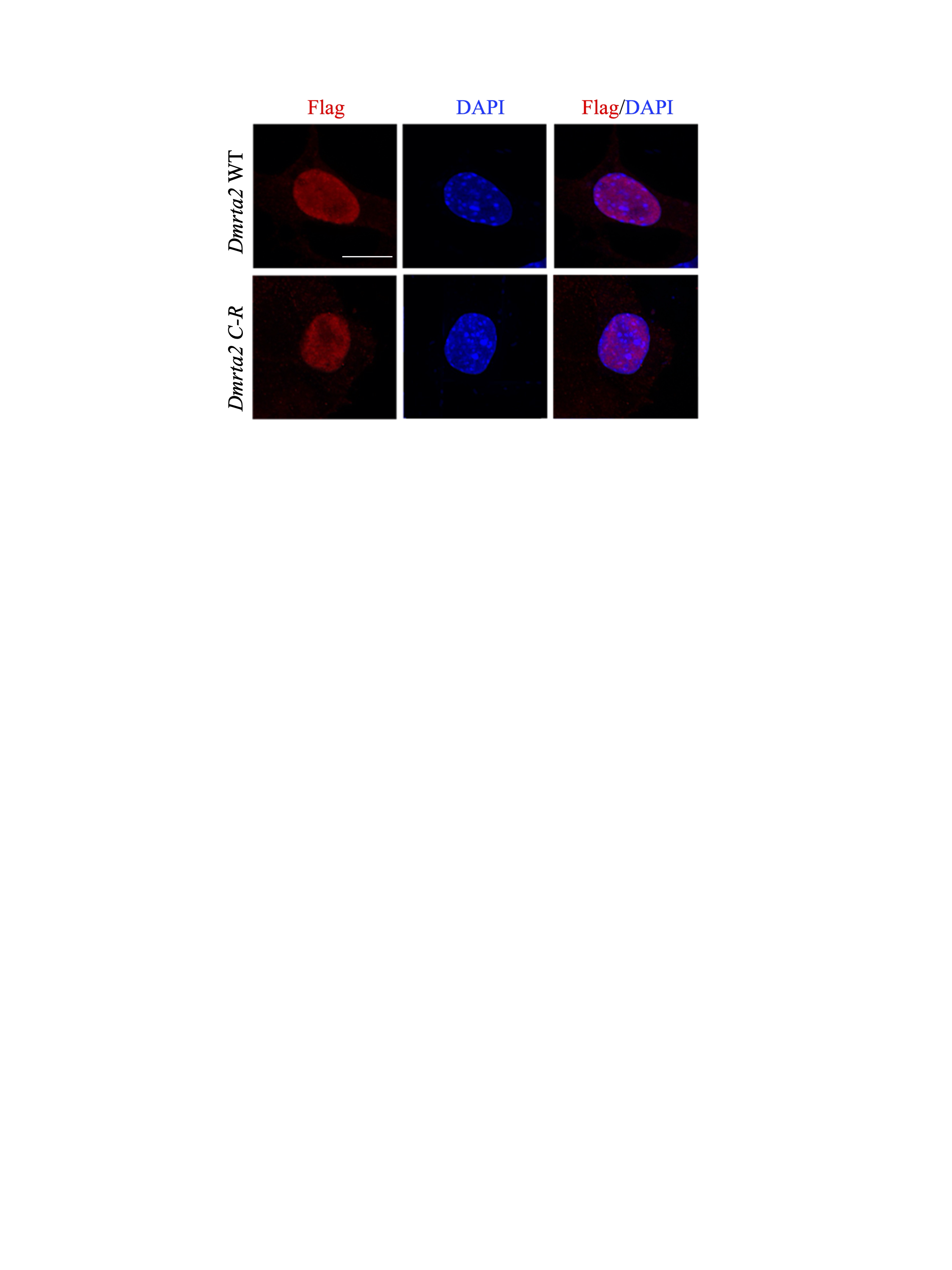

Supplement: Figure 3-2 — The Flag-Dmrta2 C-R is detected in the nucleus as the WT protein in transfected P19 cells. Flag immunostaining and DAPI staining are shown, together with a merged image. Scale bar, 10 μm. Download Figure 3-2, TIF file. [file eneuro-12-ENEURO.0377-24.2025-s007.tif]

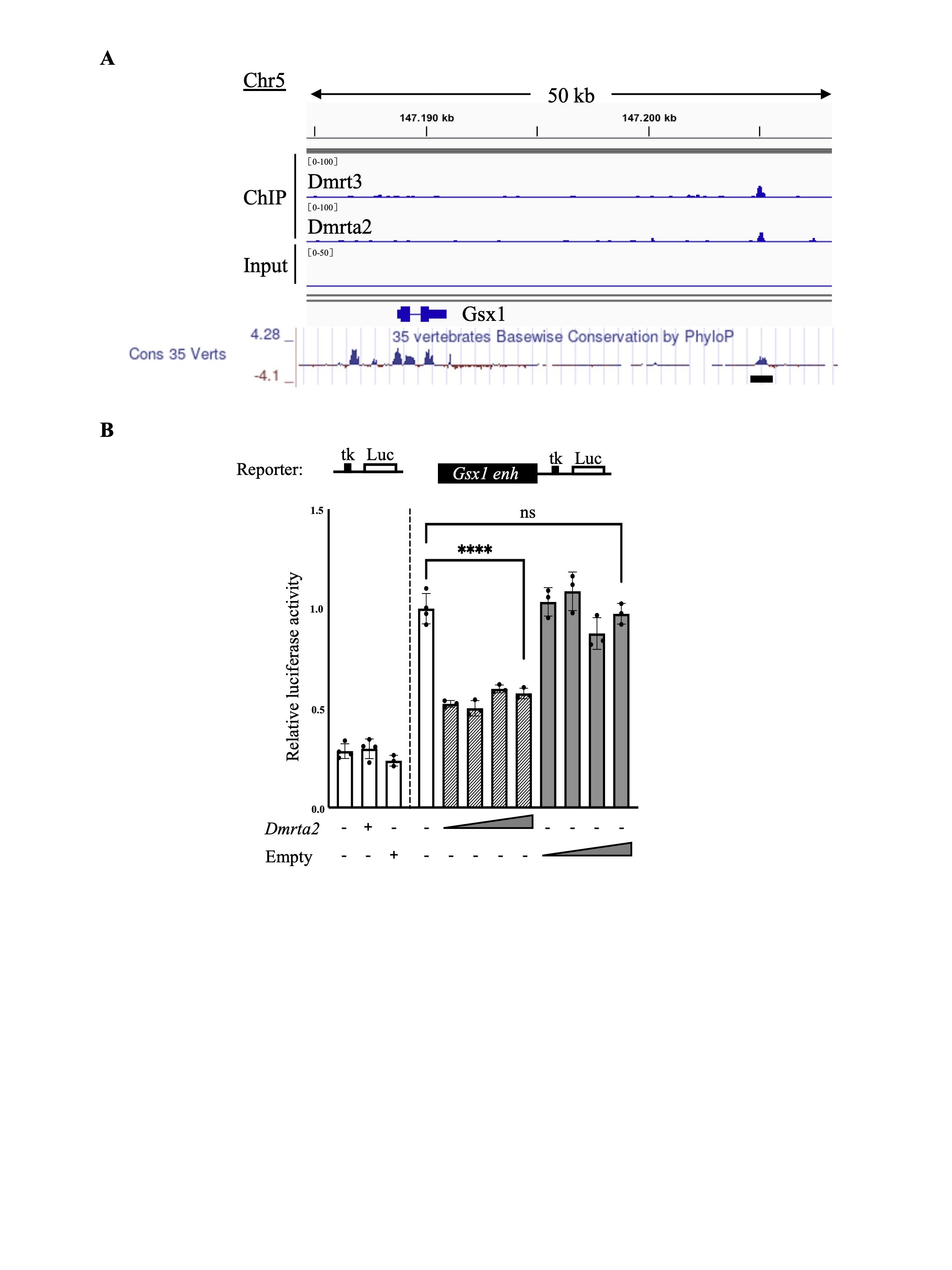

Supplement: Figure 3-3 — Dmrta2 represses the activity of an enhancer identified in the Gsx1 locus. (A) Mouse genomic region surrounding the Gsx1 gene (top) with ChIP-seq data from Konno et al., 2019 showing a conserved region bound by Dmrta2 and Dmrt3 approximately 16 kb downstream of Gsx1. (B) Reporter assays in P19 cells transfected with a tk-luc reporter with this conserved Gsx1 non-coding region upstream, or an“empty ”tk-luc reporter vector as indicated, with or without increasing amount (62.5, 125, 250, and 500 ng) of Dmrta2 or a pCS2 “empty ” plasmid. Ns: not significant, ***P < 0.0001, one-way ANOVA test. Download Figure 3-3, TIF file. [file eneuro-12-ENEURO.0377-24.2025-s008.tif]

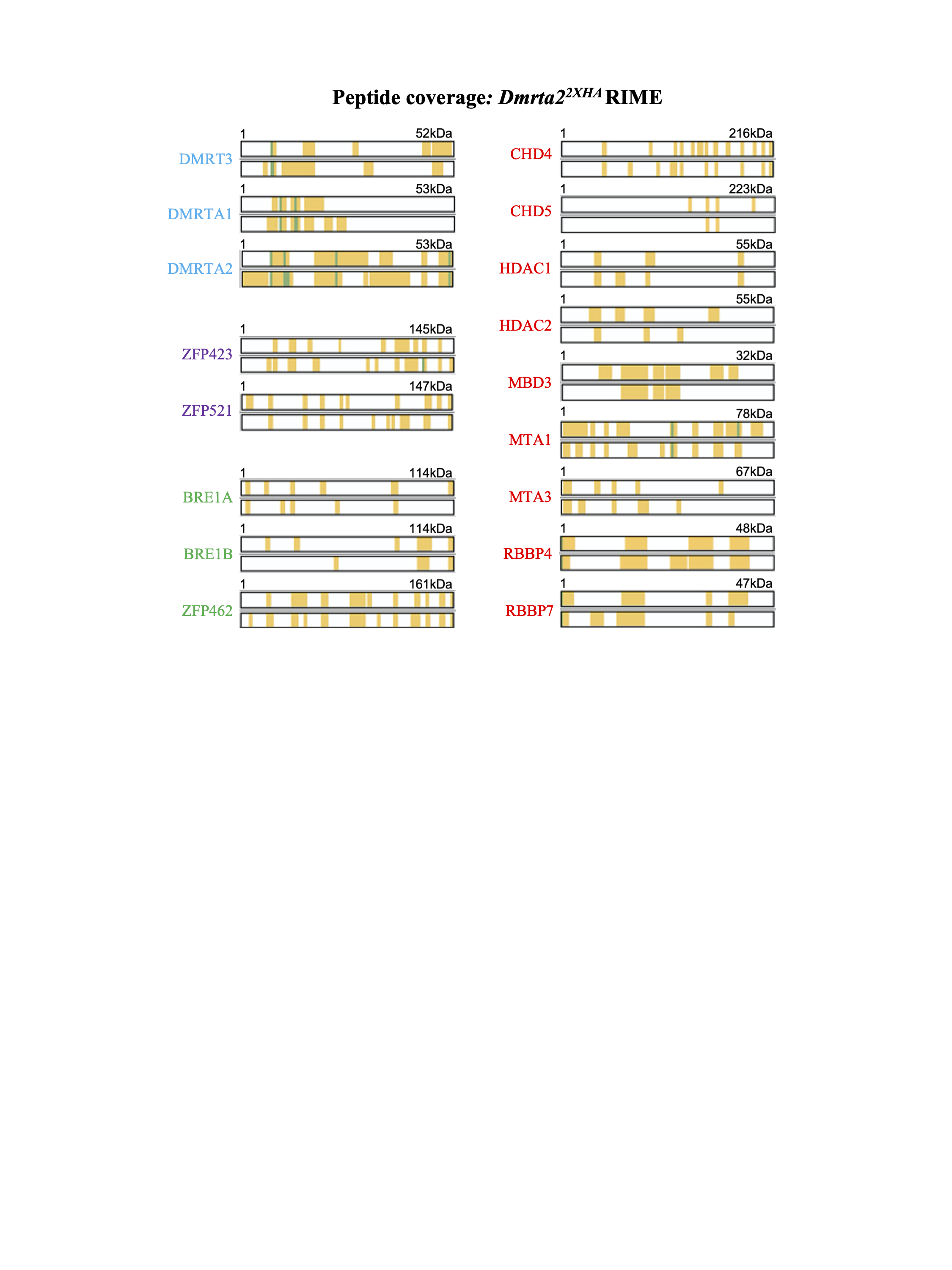

Supplement: Figure 5-1 — Peptide coverage for some of the Dmrta2 interacting proteins identified with a significance level of P < 0.05 in Dmrta22XHA embryos. Yellow bars represent regions of the full-length protein sequence where peptides were identified, n=2 for each condition. Download Figure 5-1, TIF file. [file eneuro-12-ENEURO.0377-24.2025-s010.tif]

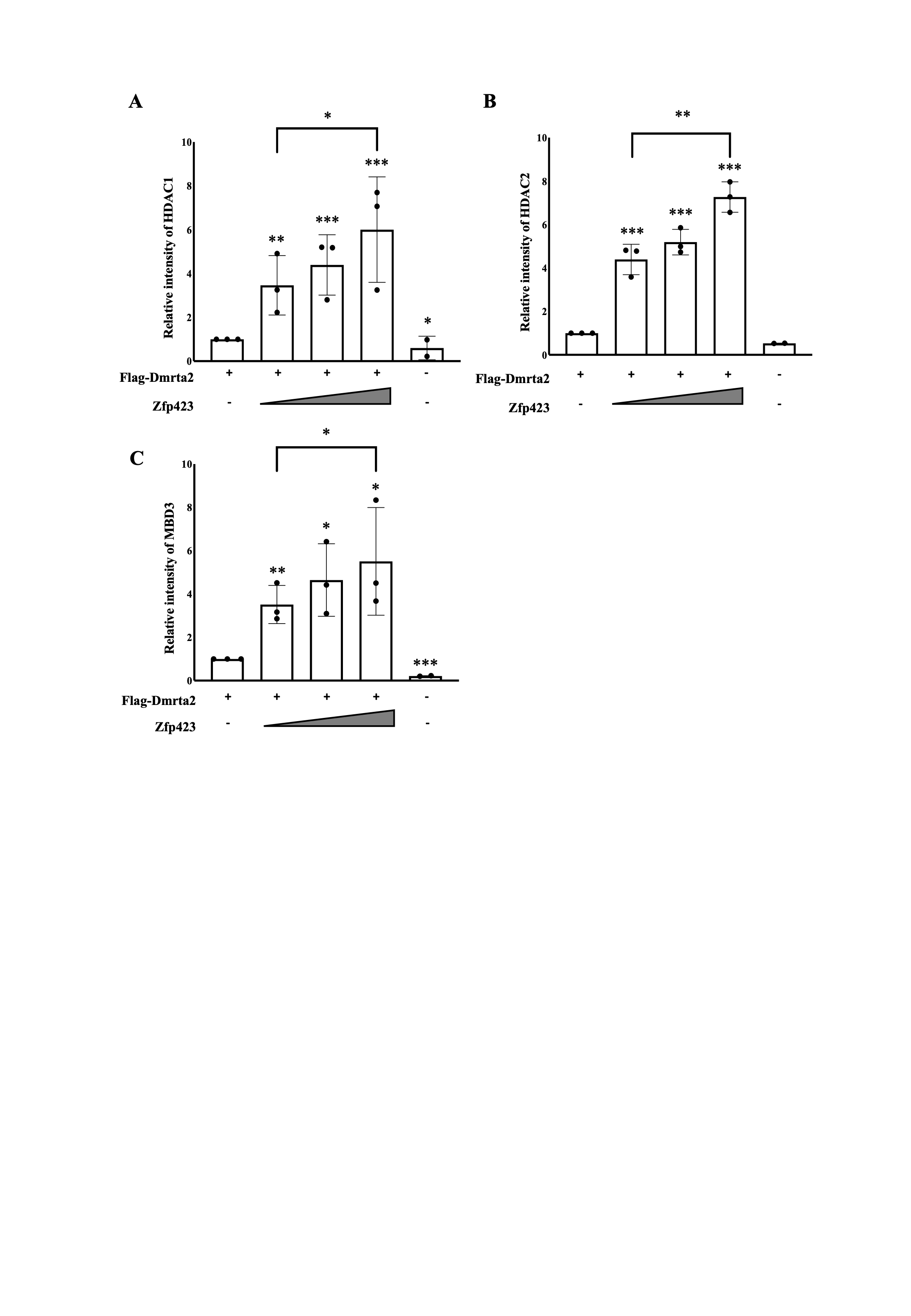

Supplement: Figure 6-1 — Densitometric quantification of Western blot results of HDAC1 (A), HDAC2 (B), and MBD3 (C) from Figure 9D. For each comparison, the value for the expression levels in cells transfected with Flag-Dmrta2 alone is set as 1. The data are presented as a comparison between these baseline cells and those transfected with additional constructs. * P < 0.05, ** P < 0.01, *** P < 0.001, one-way ANOVA test. Download Figure 6-1, TIF file. [file eneuro-12-ENEURO.0377-24.2025-s011.tif]

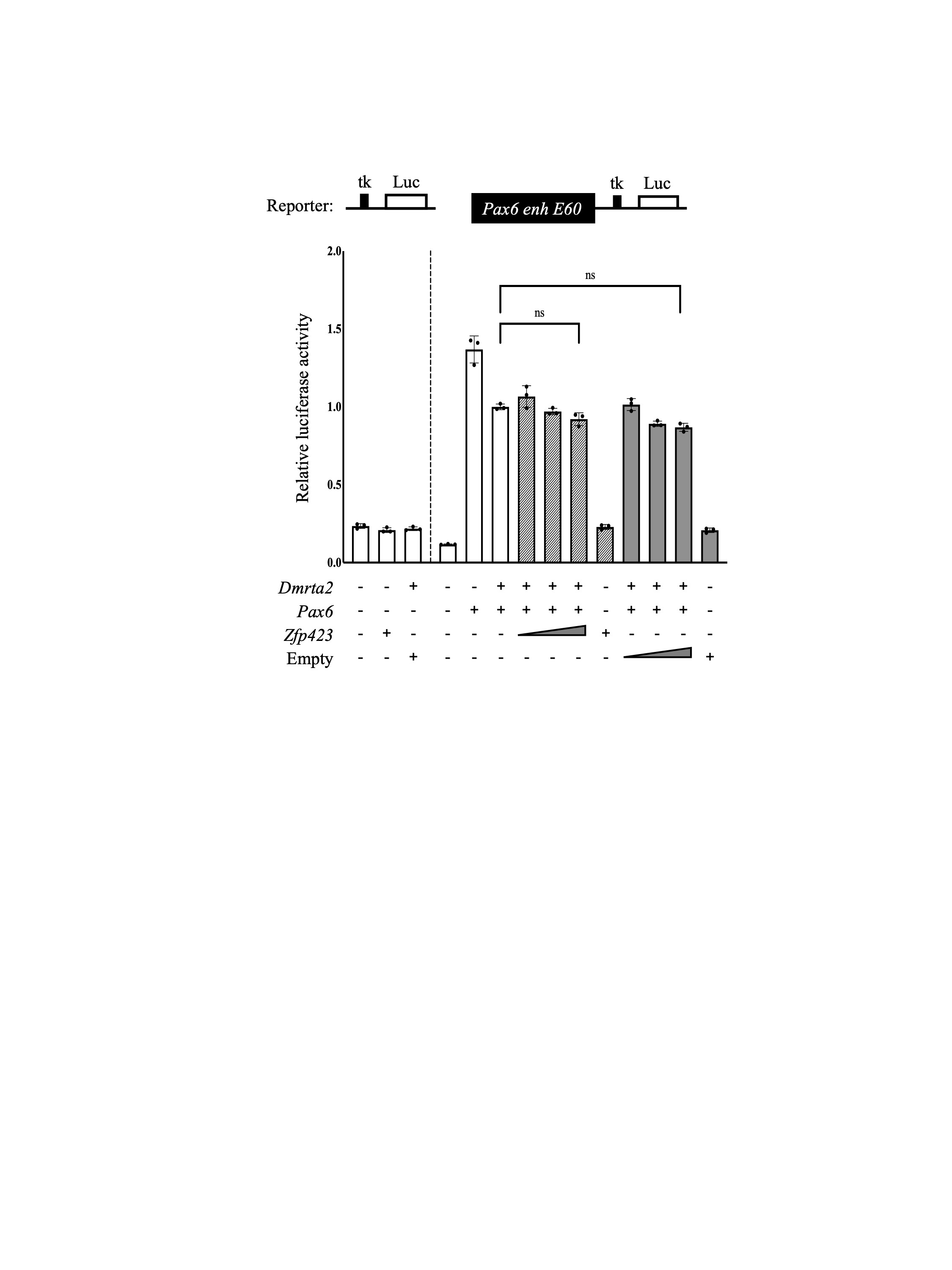

Supplement: Figure 6-2 — Zfp423 overexpression does not increase the ability of Dmrta2 to repress the activity of the Pax6 E60-tk-luc reporter in P19 cells. Reporter assays in P19 cells transfected with a Pax6 E60 tk-luc reporter vector, or an ‘empty’ tk-luc reporter vector as indicated, together with a Myc-Pax6 expression vector with or without 1ng of a Flag-Dmrta2 expression vector at as indicated, with increasing doses (from 125 ng to 500 ng) of a Myc-Zfp423 expression vector or a pcDNA3-empty vector as indicated. Values represent the mean+/- SD of one transfection done in triplicate. Ns: not significant, one-way ANOVA test. Download Figure 6-2, TIF file. [file eneuro-12-ENEURO.0377-24.2025-s012.tif]

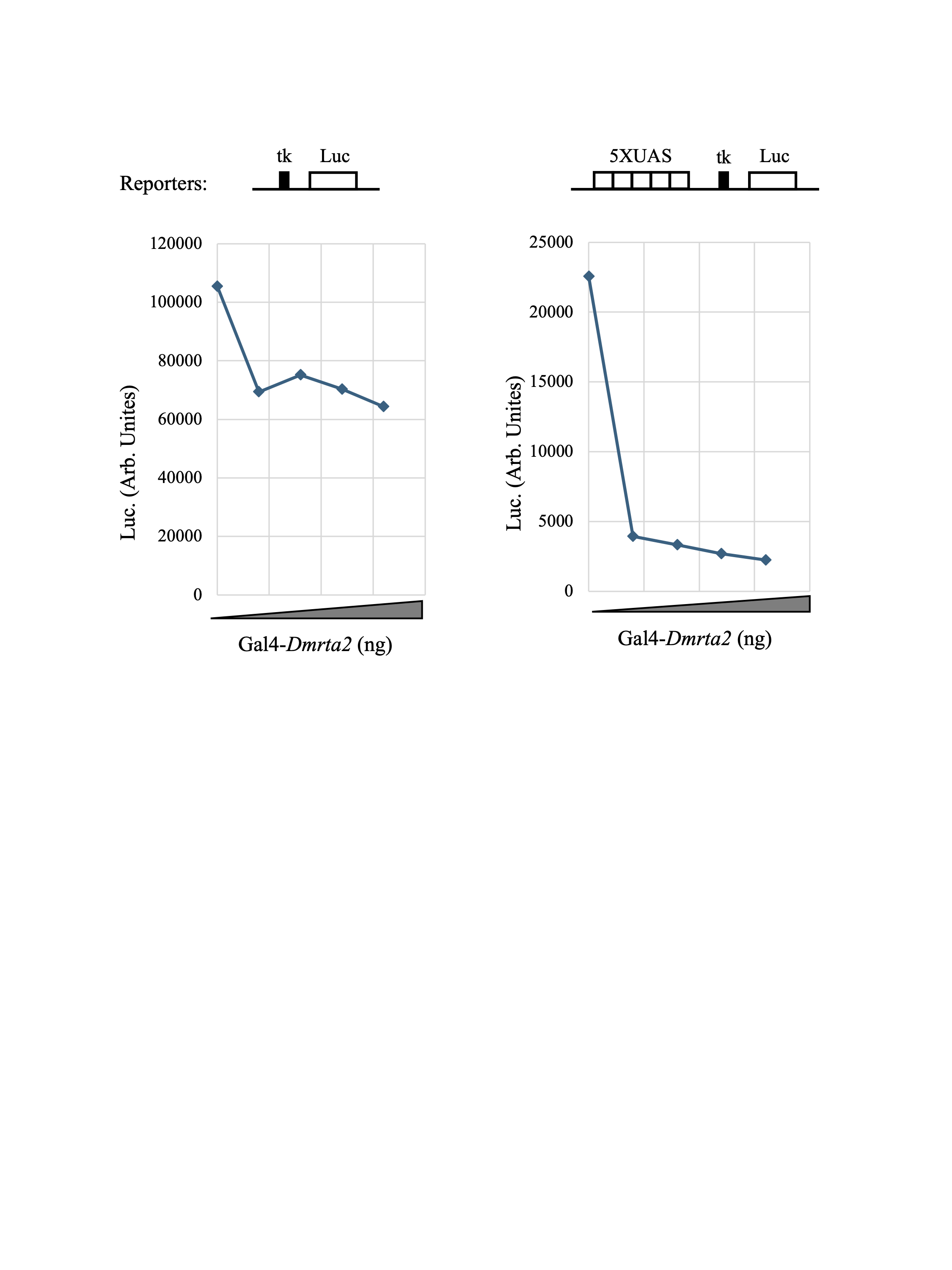

Supplement: Figure 6-3 — A Gal4-Dmrta2 fusion protein represses in a UAS-dependent manner the activity of a 5XUAS-tk-luc reporter. Results of reporter assays obtained in HEK29T3 cells transfected with an increasing dose of Gal4-Dmrta2 (50, 100, 150, and 200 ng) vector and either an “empty” tk-luc or a reporter possessing five copies of a Gal4 upstream activation sequence (5XUAS) upstream of the tk-luc reporter. Download Figure 6-3, TIF file. [file eneuro-12-ENEURO.0377-24.2025-s013.tif]

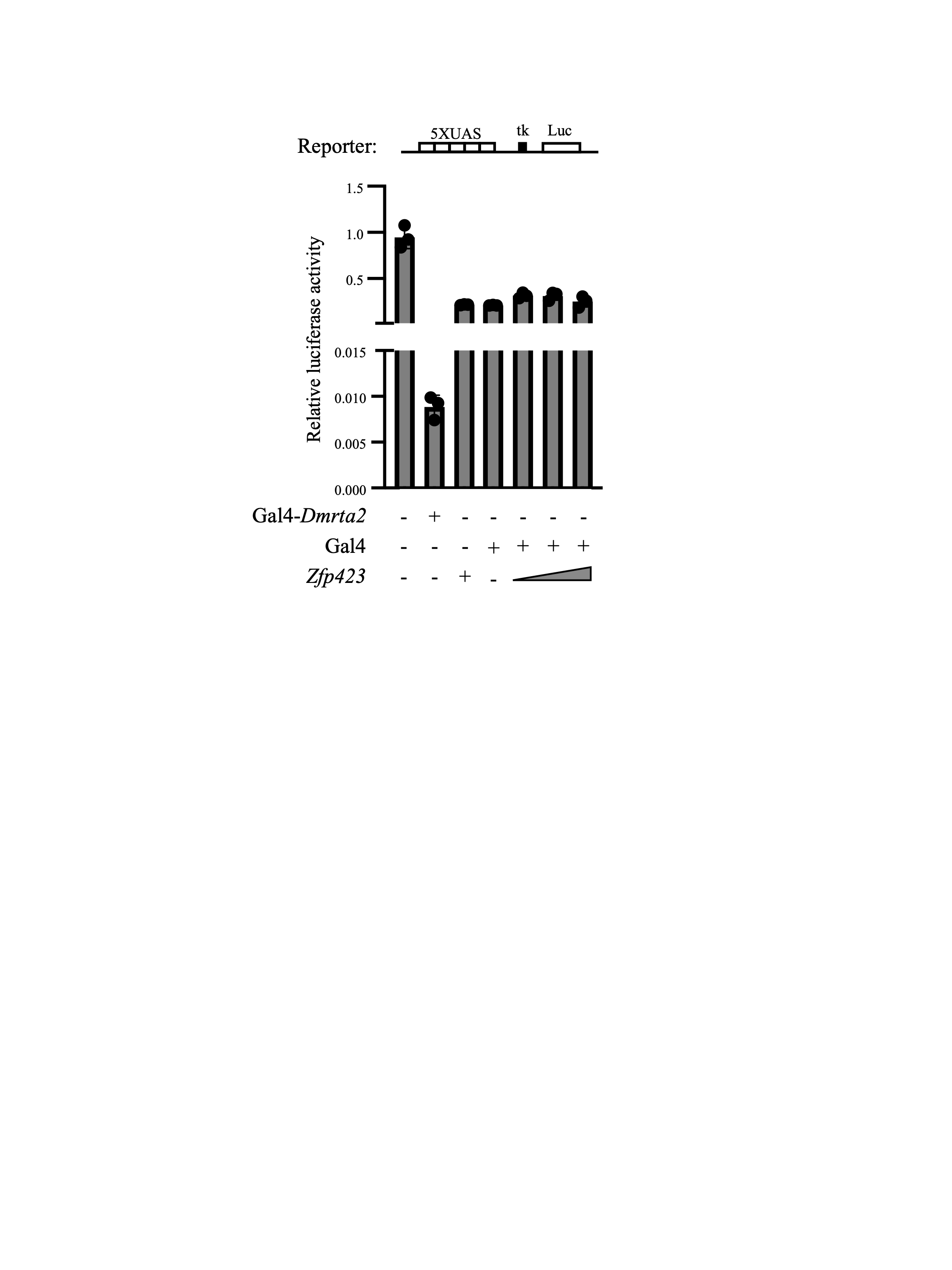

Supplement: Figure 6-4 — Reporter assays in HEK293T cells showing that Zfp423 does not increase the modest repression observed when an expression vector encoding the Gal4 DNA-binding domain alone is cotransfected with the 5XUAS-tk-luc reporter. In each condition, 200 ng of the 5XUAS-tk-luc reporter was transfected, together with 25 ng of the pCMV-Gal4-Dmrta2 or pCMV-Gal4 and different doses of pCDNA3-Myc-Zfp423 (200, 400, and 600 ng) expression plasmids. Values represent the mean+/- SD of one transfection done in triplicate. Download Figure 6-4, TIF file. [file eneuro-12-ENEURO.0377-24.2025-s014.tif]
