# Supplementary material for: Evidence That Dmrta2 Acts through Repression of Pax6 in Cortical Patterning and Identification of a Mutation Impairing DNA Recognition Associated with Microcephaly in Human
Source: eNeuro. 2025 Jun 13;12(6):ENEURO.0377-24.2025. doi: 10.1523/ENEURO.0377-24.2025 (PMC12186615; doi:10.1523/ENEURO.0377-24.2025)
Supplement: Table 4-1 — Clinical evaluation of V-3 and V-5 patients. Download Table 4-1, DOCX file. [file eneuro-12-ENEURO.0377-24.2025-s009.docx]

**Extended Data Table 6-1: Clinical evaluation of V-3 and V-5 patients.**

| **Clinical Summary of V-3** | | |
| --- | --- | --- |
| A 5-year-old boy was brought to the Neurology clinic with a history of developmental delay and excessive irritability. There is no diurnal variation. His sleep is good. He has excessive cooing spells. He has a global developmental delay, and no speech so far, but his motor milestones are getting better. He recently started crawling. No abnormal movements or posturing were observed by the parents. He smiles in return to a smile gesture. Currently, he is not taking any anti-dystonic or any muscle relaxant medications. | | |
| **Birth History:** | Full-term birth, Birth weight not known. There were no antenatal issues. C- section delivery (due to premature rupture of membrane). No history of birth asphyxia, neonatal jaundice, or feeding problems. | |
| **Developmental History:** | Delayed development. Neck holding achieved at 1.5 years, sitting without support at 2.5 years, never walked without support. Cruising started at 5 years of age No speech, he has only cooing sounds for needs. Poor cognition. He does not recognize his parents. Smiles in return. | |
| **Family History:** | Born to consanguineous parents. | |
| **Immunization History:** | Not known. | |
| **On Examination:** | Appearance: | Pale, triangular facies, prominent cheek and depressed nasal bridge, wide nares, narrow chin, prominent ears with thick helices, small forehead, long philtrum, bilateral convergent squint with Epicanthal folds but no ptosis, aberrant teeth. Lips are normal, with no periorbital puffiness, no cleft lip or palate, and no dystonic or chorea form movements observed during examination. |
|  | Head circumference: | Microcephalic (39.5cm < 2nd centile). |
|  | Posture: | **Mild positional kyphosis**, No ataxia. |
|  | Following visual stimuli and responding to auditory stimulus. Spontaneous movement in all limbs with no deficit.  Muscle bulk reduced.  **Tone decreased in all four limbs.**  **Reflexes: +3**  Bilateral convergent squint, more on the right side but no nystagmus, no cataract. No visceromegaly or murmurs.  **No Café-olait macule or hypomelanotic lesion seen, No clino or brachy, or polydyctaly.** | |

| **Clinical Summary of V-5** | | |
| --- | --- | --- |
| A 3-year-old girl was brought to the Neurology clinic with a history of abnormal body posturing, abnormal hand movements, intermittent upward gaze deviation, and excessive irritability. These movements are persistent since late infancy. There is no diurnal variation. She has never experienced any seizure episode so far. Her conscious is intact during these movements. The movements stop only during sleep. Additionally, her movements and posturing increase when she has a fever, constipation, or becomes irritable. Currently, she is not taking any anti-dystonic or any muscle relaxant medications. | | |
| **Birth History:** | Full-term birth, Birth weight not known. There were no antenatal issues. C- section delivery (but there was no indication for the C-section as per history). No history of birth asphyxia, neonatal jaundice, or feeding problems. | |
| **Developmental History:** | Delayed development. Neck holding achieved at 1 year, sitting without support at 2 years, cannot stand with support. No speech, she has only cooing sounds for needs. Poor cognition, stranger’s anxiety at the time of examination. Only recognized parents. | |
| **Family History:** | Born to consanguineous parents. | |
| **Immunization History:** | Partial vaccination until 4.5 months of age. | |
| **On Examination:** | Appearance: | Pale, prominent eyes, depressed nasal bridge, prominent maxilla, long philtrum, large malformed ears. No periorbital puffiness, no Epicanthal folds. Lips and chin were normal appearing. She was having choreoathetoid movements of the hands and dystonic posturing. Intermittent upward gaze fixation and smiling during these episodes. |
|  | Head circumference: | Microcephalic (36.5cm < 2nd centile). |
|  | Not following visual stimuli or verbal commands. She was responding to clapping sounds.  No cataract or nystagmus.  Muscle bulk reduced.  **Reflexes: +2**  Tone: slightly increased.  No visible cranial nerve deficit.  No visceromegaly or murmurs.  **No Café-olait macule or hypomelanotic lesion seen. No clino or brachydactyly, No scoliosis, No palatal anomalies.** | |
